# Structural isomerisation affects the antitubercular activity of adamantyl-isoxyl adducts

**DOI:** 10.1080/14756366.2025.2502600

**Published:** 2025-05-21

**Authors:** Yucheng Lu, Daniel Partleton, Filibus M. Gugu, Ahmed Y. G. Alhejaili, Samuel Norris, J. Jonathan Harburn, Jason H. Gill, Jonathan D. Sellars, Alistair K. Brown

**Affiliations:** ^a^ Biosciences Institute, Faculty of Medical Sciences, Newcastle University, Newcastle upon Tyne, UK; ^b^ Department of Microbiology, Plateau State University Bokkos, Jos, Nigeria; ^c^ Chemistry, School of Natural and Environmental Sciences, Newcastle University, Newcastle upon Tyne, UK; ^d^ School of Pharmacy, Faculty of Medical Sciences, King George VI Building, Newcastle upon Tyne, UK; ^e^ Translational and Clinical Research Institute, Faculty of Medical Sciences, Newcastle University, Newcastle upon Tyne, UK

**Keywords:** Mycobacterium, adamantyl, isoxyl, SQ109, antitubercular

## Abstract

Despite efforts to discover effective treatments to eradicate tuberculosis (TB), it remains a global threat. The increase in drug-resistant bacterial species has made the discovery of new drugs highly coveted. The utilisation of previous efficacious structures is one approach that can be employed to developing novel series of compounds to combat this ever-growing problem. This study sought to re-examine two such compounds, isoxyl (ISO) and SQ109, previously shown to be efficacious in TB treatment. SQ109-ISO hybrid compounds were shown to have demonstrable activity against both drug-sensitive and drug-resistant *Mtb* whilst displaying limited toxicity *in vitro* in comparison to other antitubercular agents. Indications from our genetic and biochemical studies suggest that these structurally similar pharmacophores bind to different proteins within *Mtb*, highlighting the need for careful consideration when producing regioisomeric analogues and that the utilisation of previous efficacious structures is a valid approach to developing promising novel drugs against *Mtb*.

## Introduction

Adamantyl ureas are compounds of significant interest as a result of their potency against *Mycobacterium tuberculosis (Mtb)* in ­culture, with minimum inhibitory concentrations (MIC) below 0.1 μg/mL^1^. SQ109 (Sequella) is a notable drug candidate for the treatment of Tuberculosis (TB) which utilises a new and distinct mechanism of action^2^^,^^3^. SQ109 comprises a 1,2-ethylenediamine moiety observed in Ethambutol (EMB) and a desirable adamantyl moiety and has been shown to target the Mycobacterial membrane protein Large 3 (MmpL3) of *Mtb*^4^. MmpL3 is an essential lipid transporter which translocates trehalose monomycolates (TMM) required for the incorporation of essential mycolic acid into the *Mtb* cell wall^2^^,^^3^^,^^5^. Any advance in the development of antitubercular agents is overshadowed by the ever-pressing development and speed at which drug-resistance occurs^6^. Therefore, the utilisation of previous efficacious structures is one way to create new compounds to start mitigating this problem.

**Figure 1.**
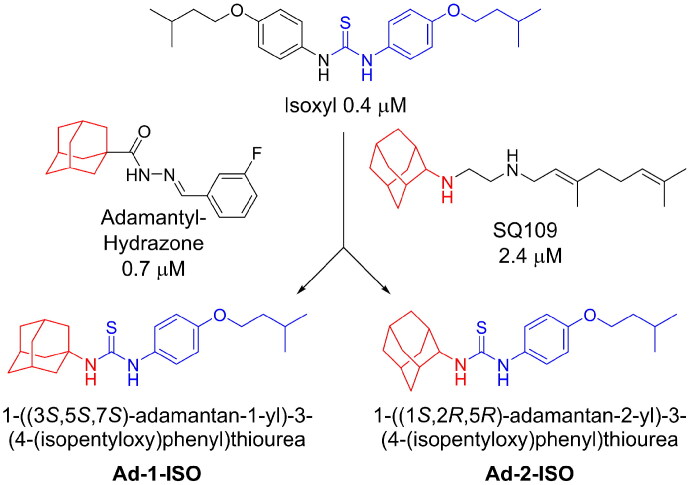
Analysis of parent drug molecules, SQ109, adamantyl-hydrazone, and ISO in the design of new hybrid molecules. ^5^^,^^25^.

Re-examination of one such compound, isoxyl (ISO) (4,4′-diisoamyloxydiphenylthiourea; 4,4′-diisoamyloxythiocarbanilide; thiocarlide), a thiourea derivative which was successfully used in the clinic during the 1960s, may provide new therapeutic targets and aid the development of novel therapeutic agents with more desirable features than earlier thioureas^7–10^. ISO was synthesised in 1953 by Buu-Hoi and Xuong as a potential anti-TB and anti-leprosy drug. Monotherapy proved to be therapeutically efficacious with 25% of patients testing sputum negative after an 8-week regimen^11^, however it is no longer used due to poor absorption in the gastrointestinal tract limiting its systemic bioavailability, affecting the overall clinical outcomes^12^. ISO, an EthA-mediated activated prodrug, is known for its bacteriostatic activity against *Mtb*^13^^,^^14^. ISO inhibits both oleic acid and mycolic acid biosynthesis in *Mtb*^2^^,^^14–16^, impacting the synthesis of species-specific lipid components of the mycobacterial membranes^17^. The stearoyl-coenzyme A desaturase, DesA3, synthesises oleic acid from stearoyl-CoA, while suppression of its activity in the presence of ISO corresponds to the depletion of tuberculostearic acid found in membrane phospholipids^18^. More recently, ISO has been shown to covalently attach to the cysteine residue (Cys61) of the HadA subunit of FAS-II dehydratase complex, inhibiting HadAB activity^15^. The HadAB-ISO complexes were devoid of dehydratase activity, resulting in the cessation of FAS-II elongation and abolished synthesis of mycolic acids^19^. Understanding the mechanism of action of old drugs and proposing chemical modifications of molecules to improve their activity and physicochemical parameters is a novel but time-consuming process^20^. A more expedient approach utilises the combination of two or more structural domains having different biological activity, creating a hybrid molecule that has the potential to act as two distinct pharmacophores^21^^,^^22^. SQ109 emerged from a similar programme of work through a combinatorial chemistry program screening 63,238 drug candidates using the 1,2-ethylenediamine pharmacophore of EMB, a first-line antitubercular agent, as its starting point^4^. In addition to *Mtb* activity, SQ109 displays efficacy against a variety of pathogens such as *Helicobacter pylori*, *Clostridium difficile*, *Neisseria gonorrhoeae and Enterococci spp*^5^^,^^23^. With the publication of the SQ109 structure, significant attention has been brought to the adamantyl group within the *Mtb* antimicrobial field^1^. It has numerous applications in drug design including increasing lipophilicity and bioavailability, modulating intramolecular reactivity and conformation, enhancing the stability and plasma half-life, and improving selectivity and potency^24^. Similarly, a library of 52,000 compounds screened against *M. bovis* BCG identified an adamantyl-hydrazone with a MIC <2 µg/mL^25^. This adamantyl-containing compound differed in its substitution position on the adamantyl ring linking via the 1-adamantyl position (tertiary, bridgehead) instead of the 2-adamantyl (secondary, bridge) position found in SQ109 (Figure 1). Therefore, given the enhanced properties that adamantyl units have on the efficacy of *Mtb* drugs coupled with the knowledge that a major issue observed with ISO was the unfavourable pharmacokinetic parameters, we sought to produce new hybrid molecules with improved antitubercular activity and increased stability.

Herein, we have evaluated two novel SQ109-ISO hybrid molecules (Figure 1), to demonstrate how the substitution position on the adamantyl ring plays a role in the antibacterial and cytotoxic activity of these molecules. Recent publications describing the SQ109-pyrrole hybrid derivatives^26^ inspired us to utilise the adamantyl pharmacophore of SQ109 (Figure 1, red) and its regioisomer seen in adamantyl-hydrazone, in combination with ISO^4^. ISO analogues have been shown to have effective antitubercular activity and are relatively easy to manipulate, therefore affording the ability to undertake an activity study of these hybrid molecules^16^.

## Materials and methods

Mycobacterial species were cultured in either Middlebrook 7H9 broth or Middlebrook 7H11 agar media supplemented with albumin–dextrose–catalase (ADC) or oleic acid–albumin–dextrose–catalase (OADC) enrichments purchased from BD Biosciences and 0.2% (v/v) glycerol, 0.2% (w/v) casamino acids, 24 μg/mL pantothenate, 200 μg/mL arginine, 50 μg/mL leucine, 1 μg/mL penicillin G, and 10 μg/mL cycloheximide, (7H9OPALPen^1^Cyc^10^). Cultures of Mycobacterial strains containing the pMV261 vector were supplemented with Kanamycin 25 μg/mL. All reagents were purchased from Sigma-Aldrich unless stated otherwise.

### Bacterial strains

BSL2 mycobacterial species used in this study were all kindly supplied by Prof. William R. Jacobs Jr (Supplementary Table 2)^27^. Non-mycobacterial strains were purchased from National Collection of Type Cultures, UK (Supplementary Table 3).

### Bacterial growth inhibition assays

Bacterial minimum inhibition concentrations (MIC) were determined using the Resazurin Microtiter Assay (REMA) method according to Palomino *et al*.^28^ Stock solutions of the tested compounds were prepared in sterile dimethyl sulfoxide (DMSO), then diluted in 7H9OPALPen^1^Cyc^10^(Kan^25^) to obtain a final drug concentration range of 32–0.032 μg/mL. A suspension of the test *Mycobacterium* was cultured in 7H9OPALPen^1^Cyc^10^(Kan^25^) containing 0.05% v/v Tween^80^ for one week at 37 °C, 5% CO_2_. The bacterial suspension was adjusted to 0.5 McFarland and diluted in 7H9OPALPen^1^Cyc^10^(Kan^25^) 1:25. 100 μL of the inoculum was added to each well of a 96-well microplate together with 100 μL of the compound titration. The plate was incubated at 37 °C, 5% CO_2_. After 5 days, 10.5 μL 0.1% (w/v) sterile resazurin (solubilised in sterile PBS containing 0.02% v/v Tween^80^) was added. Reduced resazurin was detected using fluorescence (Ex/Em 530/590 nm) in a FLUOstar Optima, BMG Labtech. All titre measurements were plotted in SigmaPlot^™^ and 4-parameter logistic (4PL) model regressions conducted. The relative MIC_95_ of each curve was calculated and averaged (Table 1). Testing was performed in duplicate with two independent biological repeats. Similarly, REMA assays were performed for the non-mycobacterial organisms but in the optimal media suggested by the EUCAST^29^. Though, for these cultures, dilutions were performed at 1:125 prior to inoculation of the plate and incubation for 4 h (Supplementary Table 3). Known antitubercular agents used in this study were rifampicin (RIF), isoniazid (INH), ISO, EMB, ethionamide (ETH), linezolid (LZD), delamanid (DEL) and moxifloxacin (MOX).

**Table 1.** Results of the antibacterial REMA assay against drug susceptible and resistant *Mtb*, and overexpression *Mtb* strains.

		*Mtb* MIC_95_ (µg/mL)									
		mc^2^7902	mc^2^8245	mc^2^8247	mc^2^8250	mc^2^8256	mc^2^7092				
Compound	Structure	pMV261	pMV261	pMV261	pMV261	pMV261	pMV261-*desA3*	pMV261-*hadABC*	pMV261-*mmpL3*	pMV261- *ethA*	pMV261-*pks13*
Isoxyl	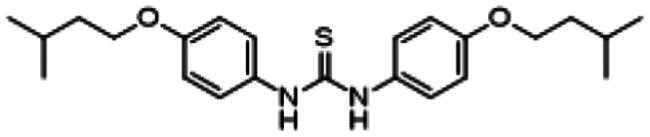	0.20	0.18	0.34	0.20	0.19	0.26	0.71	0.21	0.09	0.34
Ad-1-ISO	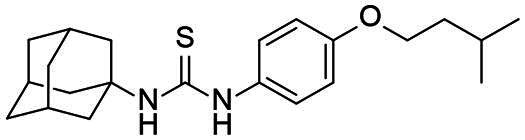	1.08	1.11	1.08	1.12	1.13	1.07	1.09	1.16	1.20	1.11
Ad-2-ISO	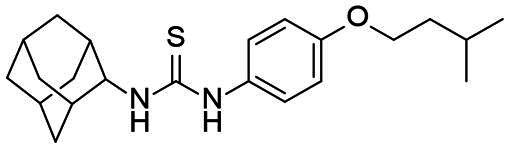	2.88	3.55	2.50	3.10	2.90	2.93	4.34	4.20	2.63	3.37
INH	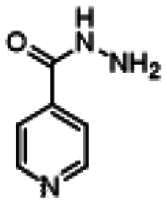	0.04	–	0.04	–	–	0.04	0.04	0.04	0.04	0.04
RIF	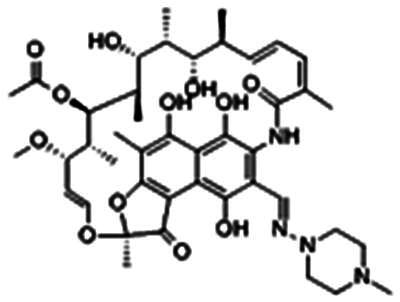	0.04	0.05	–	–	–	0.06	0.06	0.06	0.06	0.06
LZD	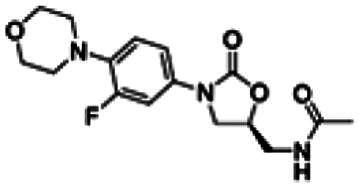	0.91	0.90	0.88	0.77	0.66	NT	0.72	0.86	NT	0.93
SQ109	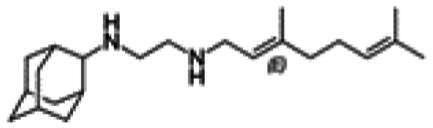	0.33	0.31	0.30	0.30	0.27	NT	0.31	0.74	NT	0.31
EMB	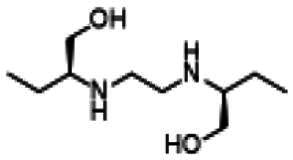	1.99	1.82	1.97	1.89	1.88	2.95	2.54	2.02	2.09	2.00
ETH	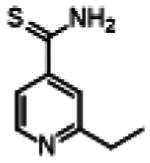	1.05	0.98	0.98	1.02	1.08	1.02	2.02	0.99	0.65	1.01

– = No activity NT = Not tested.

### Determination of cytotoxicity of compounds against mammalian cells

All compounds were tested in octuplet and against 3 independent biological repeats. RAW264.7 cell lines were purchased from American Type Culture Collection (ATCC TIB-71^™^), Human hepatoma cell line HepG2 were obtained from American Type Culture Collection (ATCC HB-8065^™^), and human cardiomyocyte cell line AC10 were obtained Dr J.H. Gill’s culture collection^30^. RAW264.7 and HepG2 cells were cultured in Dulbecco’s Modified Eagle’s Medium – high glucose (DMEM-HG) whilst AC10 cells were grown in Dulbecco’s Modified Eagle’s Medium/Nutrient Mixture F-12 (DMEM-F12). Growth media was replaced every 2–3 days and the cells washed with Hanks’ Balanced Salt Solution (HBSS) after the removal of old media and before the addition of fresh media. Once the cell culture was grown to confluence, cells were lifted with Accutase (RAW264.7) or trypsin/EDTA (AC10 & HepG2) and harvested for subculturing. Cells were cultured in the correct growth medium supplemented with 10% (v/v) foetal bovine serum (FBS), 2 mM L-glutamine and 1% (w/v) penicillin/streptomycin, at 37 °C in a humidified incubator containing 5% CO_2_. Cells were passaged after reaching 90% confluence. Cells in logarithmic growth were plated in 96-well plates at a density of 1 × 10^4^ cells/well in 200 µL of media and incubated for 24 h. The media was removed and replaced with 180 µL of fresh media. To each respective well, 20 µL of test compound at a concentration of 1 mM was added, resulting in a final drug concentration of 100 µM. In all wells the final concentration of DMSO did not exceed 0.1%. Following incubation for a further 96 h at 37 °C in a humidified incubator containing 5% CO_2_, all media was removed and replaced with 100 µL of fresh media. To each well, 20 µL of 3-(4,5-dimethylthiazol-2-yl)-5-(3-carboxymethoxyphenyl)-2-(4-sulfo-phenyl)-2H-tetrazolium, inner salt (MTS) assay solution (Promega, Madison, WI, USA) was added and incubated in the dark at 37 °C for 4 h. The absorbance of each well was recorded at 490 nm using a microplate reader, FLUOstar Optima, BMG Labtech. The percentage of cell viability was calculated relevant to the control.

### Synergy assays

Drug combination experiments were performed by two-drug assays in 96-well clear flat bottom plates. The known TB antibiotics were diluted horizontally 2.0-fold in quadruplet. Early-logarithmic cultures of *Mtb* were prepared as per the Bacterial Growth Inhibition Assays above with the addition of MIC and sub-MIC concentrations of ISO, Ad-1-ISO and Ad-2-ISO prior to inoculation of the base plate. Plates were incubation at 37 °C, 5% CO_2_ for 7 days prior to the addition of 10.5 μL 0.1% (w/v) sterile resazurin (solubilised in sterile PBS containing 0.02% v/v Tween^80^). Reduced resazurin was detected using fluorescence (Ex/Em 530/590 nm) in a FLUOstar Optima, BMG Labtech. All titre measurements were plotted in SigmaPlot^™^ and 4-parameter logistic (4PL) model regressions conducted. Synergistic, indifferent, or antagonistic interactions for various drug combinations were determined by calculating fractional inhibitory concentration (FIC) and fractional inhibitory concentration index (FICI) using the below mentioned formula as described earlier^31^.

∑FICI=FIC(A)+FIC(B)

where,

$$\text{FIC}\left(A\right)=\frac{\text{MIC}\left(A\right)\text{ in combination}}{\text{MIC}\left(A\right)\text{ alone}}$$

$$\text{FIC}\left(B\right)=\frac{\text{MIC}\left(B\right)\text{ in combination}}{\text{MIC}\left(B\right)\text{ alone}}$$

### Accumulation and extrusion inhibition

The assessment of accumulation and extrusion of EtBr by *Mtb* mc^2^7902 was performed using the method described by Rodrigues *et al*.^32^^,^^33^ *Mtb* mc^2^7902 pMV261 was grown in 20 mL of 7H9OPALPen^1^Cyc^10^Kan^25^ medium at 37 °C to an OD_600nm_ of 0.8. Cultures were centrifuged at 4,000 x *g* for 10 min, the supernatant discarded, and the pellet washed twice in 20 ml PBS-0.05% (v/v) Tween^80^ (pH 7.4).

#### Accumulation assay

10 mL of bacterial suspension was adjusted to OD_600nm_ 0.4 with PBS. 0.4% (w/v) glucose was added to a 1 mL aliquot. 90 µL of bacterial suspension was distributed to sterile black 96-well plates and 10 µL EtBr added at final concentrations that ranged from 16–0.008 µg/mL. Fluorescence was detected using a POLARstar Optima, BMG Labtech (Ex/Em 530/590 nm). Fluorescence data was acquired after incubation for 60 min at 37 °C to determine the steady-state EtBr concentration. Assays were performed in octuplet. The highest EtBr concentration for subsequent tests was used based on the EtBr titration, where readable fluorescence was detected without compromising bacterial viability. The effect of ISO, Ad-1-ISO, and Ad-2-ISO on the accumulation of EtBr was determined by adding 5 µL of each compound to aliquots of 95 µL of EtBr-containing bacterial suspension. Fluorescence data was acquired after incubation for 60 min at 37 °C. Each inhibitor was used at ½ the MIC to not compromise the cellular viability. Controls (a) Minimal accumulation = bacteria without glucose + compound; (b) Baseline accumulation = bacteria without glucose; (c) Maximal accumulation = bacteria with glucose; and (d) Accumulation inhibitor = bacteria with glucose + compound.

#### Efflux assay

*Mtb* mc^2^7902 pMV261 exposed to conditions that promote maximum accumulation of EtBr, EtBr at ½ MIC, and no glucose plate were incubated at 25 °C^32^^,^^33^. The EtBr loaded cells were centrifuged at 13,000 rpm for 3 min and resuspended in EtBr-free PBS containing 0.4% (w/v) glucose. After adjusting the OD_600nm_ to 0.4, 90 µL of bacterial suspension was distributed to sterile black 96-well plates, the effect of ISO, Ad-1-ISO and Ad-2-ISO on the efflux of EtBr was determined by adding 5 µL of titrated compound. Fluorescence was measured in a FLUOstar Optima, BMG Labtech (Ex/Em 530/590 nm) after incubation at 37 °C for 30 min. Assays were performed in quadruplet. Efflux activity was quantified by fluorescence data obtained under conditions that promote efflux (presence of glucose and absence of efflux inhibitor) with the data from the control in which the *Mtb* are under conditions of no efflux (no energy source). Thus, the relative fluorescence corresponds to the ratio of fluorescence that remains per unit of time, relative to the EtBr-loaded cells. Controls (a) Minimal efflux = bacteria without glucose + compound; (b) Baseline efflux = bacteria without glucose; (c) Maximal efflux = bacteria with glucose; and (d) Efflux inhibitor = bacteria with glucose + compound.

### Radioactive labelling analysis of lipids, fatty acid methyl esters, and mycolic acid methyl esters

*Mtb* mc^2^7902 pMV261 was grown to an OD_600nm_ of 1.0 and treated for 24 h at 37 °C with compounds/drugs (ISO, Ad-1-ISO and Ad-2-ISO) at 0 to 100 µg/mL. Cultures were then radiolabelled using [1,2-^14^C] acetic acid (0.5 μCi/mL, 50 to 62 mCi mmol^−1^; Perkin Elmer) and incubated at 37 °C for another 24 h. The culture was subsequently divided into three equal 5 mL aliquots. To isolate nonpolar and polar lipids, one aliquot was treated to organic extraction as reported earlier^34^^,^^35^. The samples (equal volumes, typically 50,000 cpm for the control) were applied to silica gel plates, and two-dimensional TLC was performed. TLC plates were exposed by autoradiography to Kodak BioMax MR film for 5 days. The cell wall bound mycolic acid methyl esters (MAMEs) (alpha, methoxy and keto) were isolated with alkaline hydrolysis, followed by methylation as previously described^34^^,^^35^. To isolate the combined fatty acid methyl esters (FAMEs) and MAMEs, the second aliquot was treated with alkaline hydrolysis, followed by methylation. The lipids were resuspended in 200 μL of chloroform. An aliquot of 5 μL lipid sample was removed, dried in scintillation vials, and then resuspended in 10 ml of scintillation fluid (Ecoscint A; National Diagnostics) and counted on a Tri-Carb 2700TR liquid scintillation analyser. To resolve FAMEs/MAMEs, equal counts of samples (∼50,000 cpm) were loaded to silica gel plates, where one-dimensional silica TLC and two-dimensional Ag^2+^ silica TLC was performed as previously^36^. The TLC plate was processed as described above, and the results were compared to recognised standards.

### Docking studies

Key mycobacterial targets identified by Deb *et al*.^37^ deemed essential for bacterial growth and 3D structures available were used in molecular docking steps. Docking of ISO and the hybrid compounds was carried out using the crystal structures of MmpL3 from *M. smegmatis* (PDB ID: 6AJG), HadAB complex from *Mtb* (PDB ID: 4RLW) and the HadBC complex from *Mtb* (PDB ID: 5ZY8), Decaprenylphosphoryl-ß-d-ribofuranose oxidoreductase (DprE1) (PDB ID: 4P8C), ß-ketoacyl acyl carrier protein synthase (FabH) (PDB ID: 1M1M), ß-ketoacyl acyl carrier protein synthase (KasA) (PDB ID: 4C6X), ß-ketoacyl acyl carrier protein synthase (KasB) (PDB ID: 2GP6), ß-ketoacyl-ACP reductase (MabA)(PDB ID: 1UZN), (3 R)-hydroxyacyl-ACP dehydratase (HadAB)(PDB ID: 7SVT), (3 R)-hydroxyacyl-ACP dehydratase (HadBC) (PDB ID: 5ZY8), Enyol-­ACP-reductase, (InhA) (PDB ID: 6R9W), Mycolic acid cyclopropane synthase (CmaA2)(PDB ID: 1KPI), Polyketide synthase (Pks13) KS Domain (PDB ID: 9F48), Pks13 AT Domain (PDB ID: 9F48), Pks13TE Domain (PDB ID: 5V40), Enoyl-CoA hydratase 6 (EchA6) (PDB ID: 5DUF), Transcriptional repressor of EthA monooxygenase (EthR) (PDB ID: 5EYR), Alanine racemase (alr) (PDB ID: 1XFC), MurE (Mur Ligase family) (PDB ID: 2WTZ), Bifunctional enzyme (GlmU) (PDB ID: 2QKX), 2-methylcitrate synthase (PrpC) (PDB ID: 3HWK), Aspartyl-tRNA Synthetase (AspS) (PDB ID: 5W25), Leucyl-tRNA synthase (LeuS) (PDB ID: 5AGS), Protein kinase B (PknB) (PDB ID: 5U94), Protein kinase A (PknA)(PDB ID: 6B2Q), Pantothenate kinase (PanK, type 1, CoaA) (PDB ID: 4BFZ), 3-pyridoxal phosphate (PLP)-dependent aminotransferase (BioA) (PDB ID: 4XJO), Aspartate aminotransferase (aspAT) (PDB ID: 6U7A) obtained from the Protein Data Bank or the EphD AlphaFold structure model (PDB ID: AF-P9WGS2-F1-v4). The proteins were loaded into SeeSAR 11.2.1^38^ and all residues within a 6.5 Å radius around the ligand, were automatically selected to use as a binding site for the docking study. The docking was carried out using FlexX integrated in SeeSAR 11.2.1^39^. 100 docking poses were generated for all compounds and the estimated affinities, torsions, and clashes calculated for all poses generated in SeeSAR analyser mode. Additionally, the bound compounds were imported into Discovery Studio (DS) 2025 (BIOVIA, Dassault Systèmes, Discovery Studio, 2025, San Diego: Dassault Systèmes, 2025). The binding sites were then defined utilising the ligands using the Define and Edit Binding Site tool generating the final structure^40^. Hydrophobicity and interactions were generated using the Display receptor surface and Display receptor-ligand interactions tools, respectively. 2-D interaction maps of the SQ109 and Ad-2-ISO with MmpL3 were generated and amino acid residues coloured according to the type of interaction formed.

### ADME prediction

An ADME (adsorption, distribution, metabolism, and excretion) prediction was performed *in silico* using the web tool SWISS-ADME (https://www.swissadme.ch).

### Adamantyl-isoxyl hybrid synthetic route

**Scheme 1.**
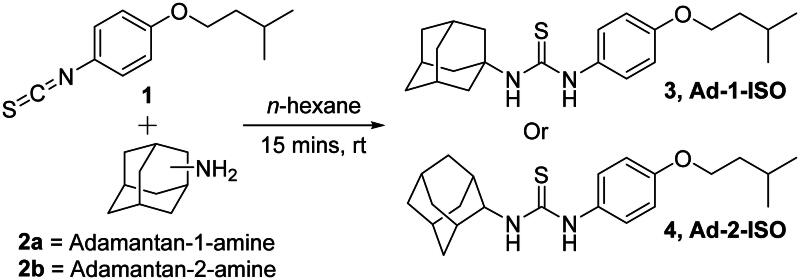
Synthesis of Ad-1-ISO and Ad-2-ISO compounds.

The hybrid molecules were synthesised using previously published methods to produce adamantyl-thiourea containing compounds and asymmetric isoxyl analogues^16^^,^^41–43^. The adamantyl compounds **3** and **4** were synthesised by reacting the arylisothiocyanate **1** with the corresponding adamantyl amine **2a** or **2b**. The arylisothiocyanate one was synthesised following established procedures, briefly comprising the alkylation of nitrophenol, followed by reduction of the nitro moiety to the aryl amine and conversion to the isothiocyanate in 20% yield over three steps^43^^,^^44^. Reaction of the arylisothiocyanate **1** with **2a** and **2b** proceeded smoothly in n-hexane, precipitating analytically pure **3** and **4** as white solids after 15 min in 54% and 31% yield respectively (Scheme 1).

#### 1-(Isopentyloxy)-4-isothiocyanatobenzene, 1

A mixture of 4-(Isopentyloxy)aniline (0.24 g, 1.34 mmol), CS_2_ (2.5 equiv.) and potassium carbonate (1 equiv.) were added to water (5 mL) and stirred overnight at room temperature. Sodium persulfate (1 equiv.), potassium carbonate (1 equiv.) and water (5 mL) were added, and this was left to stir for another hour. Brine (4 mL) was added and extracted with petroleum ether (3 × 5 mL). The organic layers were combined, dried over MgSO_4_, filtered, concentrated and dried *in vacuo*. The crude compound was then filtered through a short column of silica gel with 40–60 pet. ether to afford the title compound as a white solid (25 mg, 36%); R_f_ 0.38 (*n*-hexane); m.p. 63 – 68 °C; *ν*_max_ C-H (2957), 2869 (C-H), 2113 (N = C=S), 1501 (C = C) cm^−1^; δ_H_ (300 MHz, CDCl_3_) 7.15 (2H, d, *J* 9, Ar-*H*), 6.84 (2H, d, *J* 9, Ar-*H*), 3.97 (2H, t, *J* 7, OC*H*_2_CH_2_CH(CH_3_)_2_), 1.82 (1H, sept, *J* 7, OCH_2_CH_2_C*H*(CH_3_)_2_), 1.67 (2H, q, *J* 7, OCH_2_C*H*_2_CH(CH_3_)_2_), 0.96 (6H, d, *J* 7, OCH_2_CH_2_CH(C*H*_3_)_2_); δ_C_ (300 MHz, CDCl_3_) 158.2 (Ar-*C*), 126.9 (Ar-*C*), 123.3 (Ar-*C*), 115.3 (Ar-*C*), 66.8 (O*C*H_2_CH_2_CH(CH_3_)_2_), 37.8 (OCH_2_*C*H_2_CH(CH_3_)_2_), 25.0 (OCH_2_CH_2_*C*H(CH_3_)_2_), 22.6 (OCH_2_CH_2_CH(*C*H_3_)_2_); *m/z* (ES^+^) 254 (M + MeOH + H^+^) and is commercially available.

#### 1-((3s,5s,7s)-adamantan-1-yl)-3–(4-(isopentyloxy)phenyl)thiourea, 3 [ad-1-ISO]

A solution of 1-(isopentyloxy)-4-isothiocyanatobenzene (21 mg, 0.11 mmol) in n-hexane (3 mL) was treated with 1-(Isopentyloxy)-4-isothiocyanatobenzene (1 equiv.) and stirred at room temperature. A white solid precipitated after 15 min, which was filtered and washed with n-hexane (3 × 3 mL) to afford the title compound as a white solid (22 mg, 54%); R_f_ 0.57 (*n*-hexane/EtOAc [3:1]); m.p. 133 – 137 °C; *ν*_max_ 3337 (N-H), 3261 (N-H), 2898 (C-H), 2852 (C-H), 1538 (C = C), 1510 (C = C), 1222 (C = S) cm^−1^; δ_H_ (300 MHz, CDCl_3_) 7.10 (2H, d, *J* 9, Ar-*H*), 6.90 (2H, d, *J* 9, Ar-*H*), 5.96 (1H, bs, N*H*), 3.97 (OC*H*_2_CH_2_CH(CH_3_)_2_), 2.20 − 2.15 (6H, m, Ad-*H*), 2.11–2.06 (3H, m, Ad-*H*), 1.83 (1H, sept., *J* 7, OCH_2_CH_2_C*H*(CH_3_)_2_), 1.70 − 1.63 (8H, m, Ad-*H*, OCH_2_C*H*_2_CH(CH_3_)_2_), 0.96 (6H, d, *J* 7, OCH_2_CH_2_CH(C*H*_3_)_2_); δ_C_ (300 MHz, CDCl_3_) 179.3 (NH*C*(S)NH), 158.5 (Ar-*C*), 128.8 (Ar-*C*), 127.7 (Ar-*C*), 115.9 (Ar-*C*), 66.8 (O*C*H_2_CH_2_CH(CH_3_)_2_), 54.6 (Ad-*C*), 41.7 (Ad-*C*), 38.0 (OCH_2_*C*H_2_CH(CH_3_)_2_), 36.4 (Ad-*C*), 29.7 (Ad-*C*), 25.2 (OCH_2_CH_2_*C*H(CH_3_)_2_), 22.7 (OCH_2_CH_2_CH(*C*H_3_)_2_); *m/z* (ES^+^) 373 (MH^+^); HRMS (ES^+^) Found MH^+^, 373.2323 (C_22_H_33_N_2_OS requires 373.2314).

#### 1-((1r,3r,5r,7r)-adamantan-2-yl)-3–(4-(isopentyloxy)phenyl)thiourea, 4 [ad-2-ISO]

Adamantan-2-amine hydrochloride (0.1 g, 0.53 mmol) was dissolved in water (3 mL) and treated with aq. NaOH (1 equiv., 1 M) at room temperature. A precipitate formed, stirred for 10 min and then extracted with n-hexane (3 × 5 mL). The resultant n-hexane solution was treated with a solution of 1-(isopentyloxy)-4-isothiocyanatobenzene (0.13 g, 0.59 mmol) at room temperature. After stirring for 15 min a white precipitate was filtered, washed with n-hexane (3 × 3 mL), and dried to afford the title compound as a white solid (62 mg, 31%); m.p. 130 – 132 °C; *ν*_max_ (ATR) 2912, 2851, 1540 (C = S), 1508, 1475, 1370, 1359, 1281, 1237, 1166, 1100, 1056 cm^−1^; δ_H_ (400 MHz, CDCl_3_) 7.38 (1H, bs, N*H*), 7.15 (2H, d, *J* 8, Ar-*H*), 6.95(2H, d, *J* 8, Ar-*H*), 6.25 (1H, d, *J* 7, N*H*), 4.44 (1H, bs, Ad-*H*), 4.00 (2H, t, *J* 7, OC*H*_2_CH_2_CH(CH_3_)_2_), 2.07 (2H, bs, Ad-*H*), 1.90 − 1.64 (11H, m, OCH_2_C*H*_2_C*H*(CH_3_)_2_, Ad-*H*), 1.60 (2H, m, Ad-*H*), 1.37 (2H, m, Ad-*H*), 0.97 (6H, d, *J* 7, OCH_2_CH_2_CH(C*H*_3_)_2_); δ_C_ (100 MHz, CDCl_3_) 179.8 (NH*C*SNH), 158.2 (*ipso*-Ar-*C*), 128.4 (*ipso*-Ar-*C*), 127.5 (Ar-*C*), 115.9 (Ar-*C*), 66.8 (O*C*H_2_CH_2_CH(CH_3_)_2_), 58.7 (Ad-*C*), 37.9 (OCH_2_*C*H_2_CH(CH_3_)_2_), 37.4 (OCH_2_CH_2_*C*H(CH_3_)_2_), 36.9 (Ad-*C*), 32.4 (Ad-*C*), 31.6 (Ad-*C*), 27.01 (Ad-*C*), 26.98 (Ad-*C*), 25.1 (Ad-*C*), 22.6 (OCH_2_CH_2_CH(*C*H_3_)_2_); *m/z* (ES^+^) 373 (MH^+^); HRMS (ES^+^) Found MH^+^, 373.2318 (C_22_H_33_N_2_OS requires 373.2314).

## Results

### Activity of hybrid molecules against mtb

The generated hybrid compounds and a variety of antitubercular agents were tested against a panel of *Mtb* strains including drug-sensitive, drug-resistant and gene overexpression strains. The overexpression strains were selected based on their indicated role in the mode of action of either SQ109 or ISO. SQ109 has emerged as a promising drug against a variety of bacterial pathogens^5^^,^^23^. Whereas, ISO showed no activity against the ESKAPEE pathogens (Table S4) and is relatively bacteria-specific, targeting only *Mycobacterium sp*. The hybrid molecules retained this species specificity observed with ISO, showing no activity against the ESKAPEE pathogens (Table S4) whereas they demonstrated potent inhibition of drug-sensitive and drug-resistant *Mtb* strain growth (Table 1). The overexpression of DesA3 in *Mtb* mc^2^7902 (Wild type, WT) did not confer any resistance to the compounds tested. Even ISO resistance was not observed which was unexpected. The MIC of ISO against *M. bovis* BCG was previously determined by microplate alamar blue assay to be 3.0 µg/mL and 6.0 µg/mL when *M. bovis* BCG contained the pVVdesA3 overexpression plasmid^14^. As this result was quite unexpected we were pleased to observe that the overexpression of the HadABC complex conferred resistance to ISO as previously reported^19^^,^^45^. Interestingly, overexpression of the HadABC complex conferred resistance to Ad-2-ISO but not Ad-1-ISO suggesting a possible role for these essential proteins in the mode of action. As previously observed MmpL3 has been confirmed as a target of SQ109 and its overexpression conferred partial resistance in our study^46^. Both Ad-2-ISO and SQ109, but not Ad-1-ISO or ISO, were shown to be perturbed in the single drug assays through the overexpression of MmpL3. This observation is suggestive of a similar mode of action for our Ad-2-ISO hybrid as SQ109 targeting the MmpL3 protein in *Mtb*. As ISO is a prodrug activated by EthA-mediated oxidation via the thiourea, we sought to assess the effects of EthA overexpression on our hybrid compounds. EthA overexpression and the activation of ISO was again confirmed as previously demonstrated, however, no change in the MIC was observed in the hybrid compound tests^47^. Therefore it can be assume that converse to ISO, the hybrid compounds are not EthA-mediated prodrugs. Enzymes involved in late-stage mycolic acid synthesis are keen areas of interest, therefore the compounds were tested against *Mtb* overexpressing Polyketide synthase 13 (Pks13) ^48^. Pks13 of Mtb catalyses the last step of mycolic acid synthesis prior to its esterification onto trehalose to form the TMM and subsequently exported to the cell wall. No significant changes were observed in the MICs of any compound tested against this overexpression strain.

### Cytotoxicity

The cytotoxicity of anti-TB drugs and our hybrid compounds were evaluated against uninfected RAW264.7 murine macrophage cells, HepG2 human liver cancer cell line, and AC10 hybrid adult ventricular cardiomyocytes after exposure to the compounds for 96 h; cell viability was measured using standard MTS analysis.

ISO and LZD showed cytotoxicity against the uninfected RAW264.7 macrophage cells at 100 µM, 34.5% and 38.3% survival, respectively. The other known antitubercular agents demonstrated relatively low levels of toxicity against murine macrophages and other cells tested, however, SQ109 showed toxicity against the HepG2 and AC10 cells (Table 2). Moreover, somewhat disappointingly the Ad-2-ISO also had significant cytotoxicity at 100 µM against all three cell lines tested.

**Table 2.** Cytotoxicity of antitubercular agents at 100 µM.

	MTS percentage survival @ 100 µM		
	RAW264.7	HepG2	AC10
Compound	Macrophage	Liver	Cardiac
ISO	33.6 ± 3.6	95.4 ± 4.3	38.9 ± 13.9
Ad-1-ISO	73.8 ± 7.5	16.1 ± 7.2	55.0 ± 13.2
Ad-2-ISO	22.5 ± 0.9	13.4 ± 5.1	51.1 ± 17.7
INH	96.3 ± 2.0	97.0 ± 3.8	92.6 ± 1.2
RIF	66.3 ± 5.0	47.7 ± 3.2	40.7 ± 6.6
LZD	36.4 ± 1.3	105.4 ± 5.1	103.0 ± 1.8
SQ109	61.1 ± 2.9	15.1 ± 4.1	40.2 ± 2.3
EMB	88.5 ± 8.6	95.2 ± 6.0	93.7 ± 2.7
ETH	100.1 ± 5.6	92.7 ± 7.0	90.5 ± 4.7

**Figure 2.**
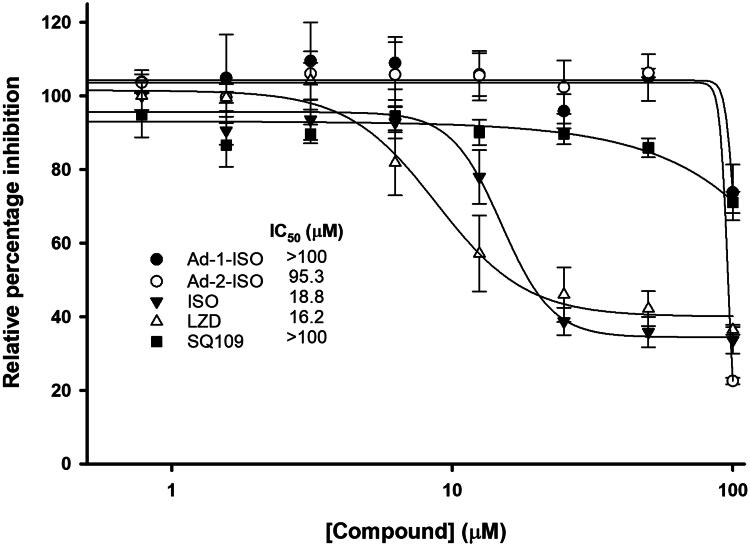
Cytotoxicity assay in murine macrophage-like cells (RAW264.7) cells. Cells were treated with the test compounds (0 to 100 μM) using DMSO as a negative control. Cell viability was measured by MTS assay and IC_50_ of compounds calculated.

To further assess the potential toxicity of these compounds half-maximal inhibitory concentration (IC_50_) were calculated for the ISO, SQ109, LZD (known toxic control) and the two adamantyl-ISO hybrid compounds were performed to investigate the drug’s efficacy and determination of a selectivity indices (SI) (Figure 2). ISO, LZD and SQ109 had IC_50_ values of 18.8, 16.2, and >100 µM, respectively against RAW264.7 macrophages.

With MIC_95_ values of 0.49 µM (0.2 µg/mL), 1.63 µM (0.55 µg/mL) and 0.36 µM (0.12 µg/mL), the cytotoxic activities provide us with SI’s of 38, 16 and >100 for ISO, LZD, and SQ109 respectively. Encouragingly, even with the marginally reduced potency against WT *Mtb*, in comparison to the parental compounds, the Ad-2-ISO derivative showed a comparable SI of 39. The Ad-1-ISO SI was determined to be greater than 100 indicating that these compounds could both be used in a therapeutic window like that of SQ109 and LZD. Unfortunately, this assay was limited by the maximum solubility of the compound in DMSO and the threshold of cytotoxicity of the vehicle reagent in the MTS assay. The detailed testing against these cell lines revealed that the two adamantyl-ISO derivatives were also only cytotoxic at high levels against the human-derived liver cells HepG2, and the AC10, human-derived cardiomyocyte. In comparison to ISO and SQ109, although the level of activity against *Mtb* is marginally inferior, these compounds are consistently less toxic, justifying the hybrid methodology employed in the study. The human-derived hepatoblastoma cell line, HepG2, is predominately used in antimicrobial toxicity studies to investigate the toxic and metabolic effects of the compound against cells of the hepatic system. Therefore, the toxicity observed may be indicative of the biotransformation of the thiourea within the hybrid molecules. Studies with isolated enzyme preparations have demonstrated that thiourea can be metabolised by hepatic enzymes oxygenating the lipophilic N-substituted thioureas converting them to the sulphonic acids. This metabolite is responsible for the toxicity observed as when administered alone sulphonic acid is considerably more toxic than thiourea^49^.

### Synergy determination of hybrid molecules and standard drugs against mtb

**Figure 3.**
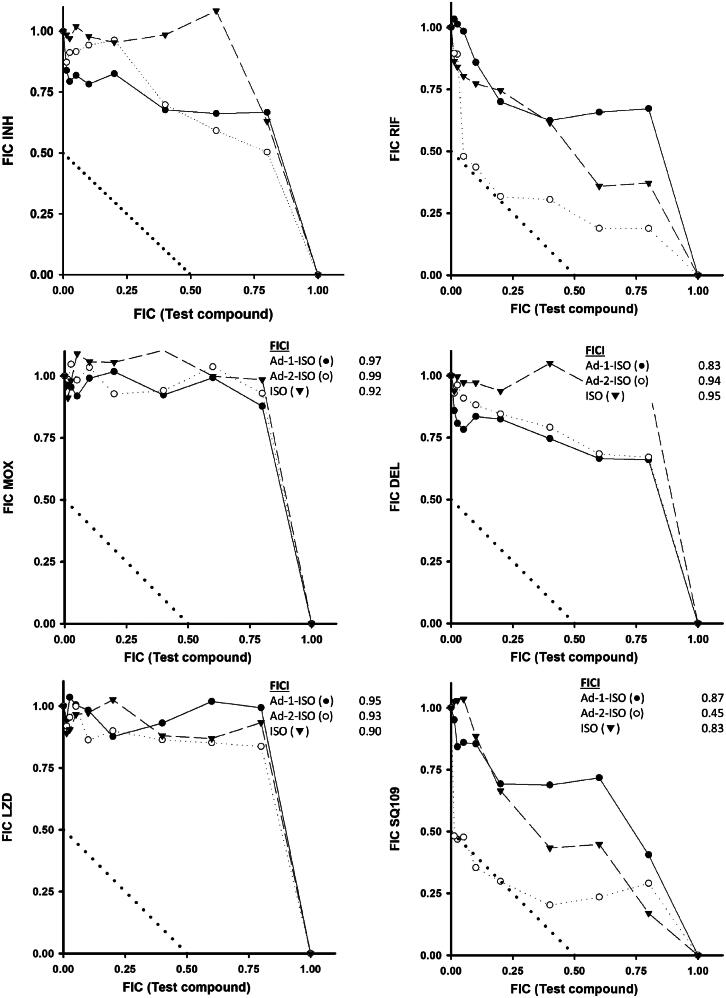
The activity profiles of Ad-1-ISO (●), Ad-2-ISO (○) or ISO (▾) in combination with INH, RIF, MOX, DEL, LZD, and SQ109 against *Mtb* mc^2^7902 as per the materials and methods. FIC values for each drug in combination with either Ad-1-ISO, Ad-2-ISO or ISO were determined and shown as isobolograms. FICI calculations for the best combination are inset in each subfigure, ‘synergy’ (FICI ≤ 0.5), ‘antagonism’ (FICI > 4.0) and ‘no interaction’ (FICI > 0.5–4.0). The isobolograms are representative of two independent experiments performed in duplicate.

The fractional inhibitory concentration index (FICI) of the two adamantyl-ISO derivatives was evaluated using a checkerboard style assay to determine whether a synergistic effect exists between the hybrid compounds and current antitubercular drugs. The Journal of Antimicrobial Chemotherapy stipulated that FICI data is restrict to interpretations of “synergy” (FICI ≤ 0.5), “antagonism” (FICI > 4.0) and “no interaction” (FICI > 0.5–4.0). The activity of the Ad-1-ISO and Ad-2-ISO in combination with RIF, INH, MOX, DEL, LZD, and SQ109, against *Mtb* mc^2^7902 pMV261 were determined (Figure 3).

Interestingly, there was a significant drop in the MIC of SQ109 in the presence of Ad-1-ISO and Ad-2-ISO. Moreover, the FICI indicated a significant synergy between SQ109 and Ad-2-ISO. This could be attributed to the similarity in structure and therefore the possible dual targeting of MmpL3, or a synergistic effect of hitting different secondary targets which results in the observed drop in the combination MIC. The FICI of this combination was calculated at 0.49, falling just within the synergy range. The combination of SQ109 and Ad-1-ISO did not result in a synergistic outcome which, as per the MIC data, is suggestive that this compound is interacting differently with its potential target protein. It is plausible that the change in the regiochemistry of the adamantyl moiety is responsible for this. Ad-1-ISO is closer in structural relationship to AU1235, a promising adamantyl urea with antitubercular activity, which has been proposed to target the epoxide hydrolase, EphD^50^^,^^51^ as well as MmpL3^2^.

As ISO was also demonstrated to target EphD, the inhibition of this key detoxifying enzyme involved in the catabolism of mycolic acids could result in negative pressure on the ability of the organism to remodel its mycolate outer membrane layer when combined with an MmpL3 inhibitor^50^^,^^51^. Interestingly, the combination of RIF and Ad-2-ISO produced a FICI of 0.52, just above the defined synergy breakpoint. Previously, synergies between RIF and hydroperoxide/antioxidants were indicative of the possible interplay of reactive oxygen species in the mode of action of these novel compounds, therefore the interplay between RIF and Ad-2-ISO warrants further investigation^52^^,^^53^. No other notable synergies were observed across the other combinations tested.

### Accumulation and extrusion inhibition

In some drug-resistant clinical strains, genetic mutations have not been found in known drug targets, but multiple efflux pumps, including EfpA and MmpL7, have been observed with significant overexpression^54–57^. This suggests that these efflux pumps may contribute to drug resistance. Previous reports showed that *efpA* expression increased in response to INH^58^^,^^59^, thiolactomycin^60^, ISO, and tetrahydrolipstatin^61^. The induction of *efpA* expression has been observed to be between 4 and 4.5-fold greater under INH stress^59^. EfpA, from the major facilitator superfamily (MFS), is highly conserved among both slow-growing and fast-growing *Mycobacterium spp*^62^. To assess the effect of our hybrid compounds we compared the rate of accumulation of ethidium bromide (EtBr) in the presence of ISO and the two adamantyl hybrid compounds as an indirect method to evaluate the efflux capacity of the *Mtb* mc^2^7902 pMV261 strain. For this purpose, we determined the relative final fluorescence (RFF) indexes. The RFF index is a measure of how effective a compound is on the inhibition of EtBr efflux (at a given concentration) by comparison of the final fluorescence at the last time point (60 min) of the treated cells with the cells in the presence of EtBr only^63^. The RFF is a measure of efflux inhibition by comparing the final fluorescence of efflux pump inhibitor treated vs untreated cells. The RFF value >1 indicates an enhanced accumulation of EtBr^64^.

**Figure 4.**
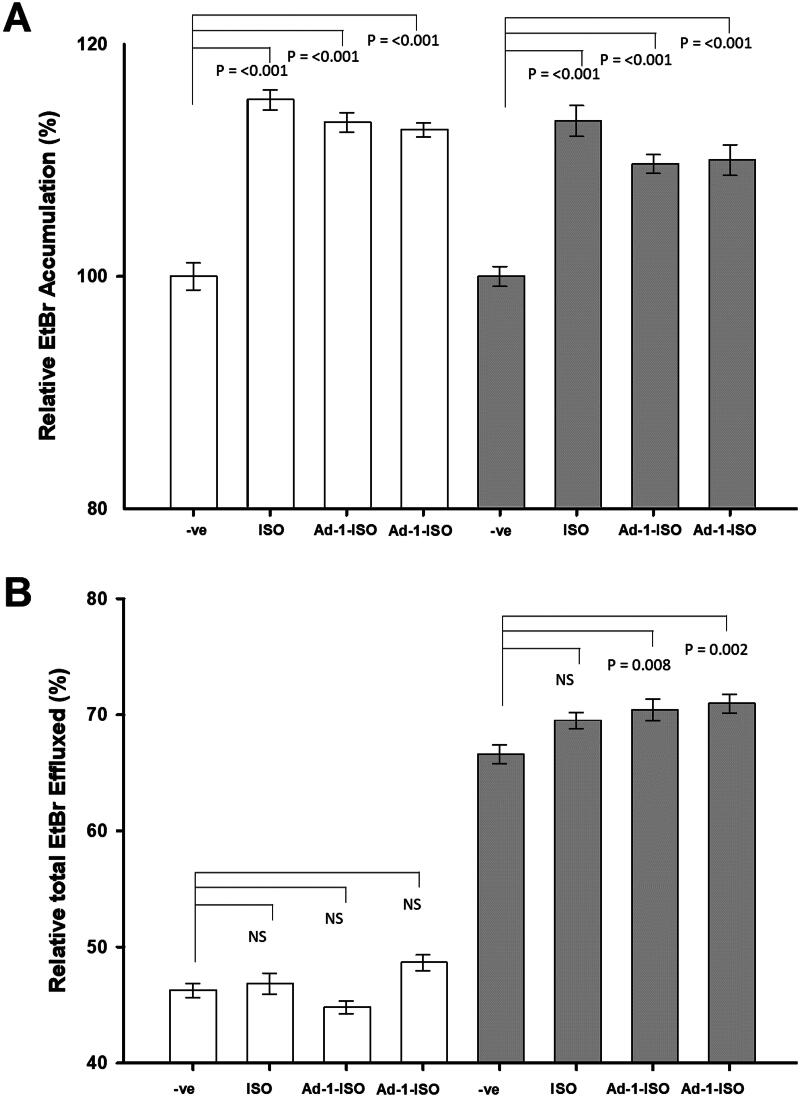
Accumulation and efflux of EtBr by *Mtb* mc^2^7902 pMV261. A) Accumulation of EtBr under conditions that maximise efflux, presence of glucose (dark grey), and without glucose (white). B) Efflux takes place at 37 °C in the presence and absence of glucose and the inhibitors. EtBr was used at ½ MIC to ensure maximum EtBr-loading of the bacteria, without compromising cellular viability. One Way Repeated Measures Analysis of Variance (ANOVA) was used to investigate the significance of the glucose (dark grey) and no glucose (white), in the presence of the inhibitor.

ISO and the two adamantyl hybrid compounds all demonstrate increased accumulation of EtBr in the absence or presence of glucose (Figure 4). Additionally, One Way Repeated Measures Analysis of Variance (ANOVA) analysis of the results indicated that there was a significant difference observed between the negative controls of each assay and assays containing our compounds, however, there was no significant difference observed when each compound was compared with and without glucose. Glucose reenergises the cells, promoting active efflux and reducing EtBr accumulation in accumulation assays, or increased efflux of EtBr in efflux assays by generating ATP energy. The combination of this known theory and our results would suggest that the compounds are affecting the efflux of EtBr during the accumulation assays, or they are actively reducing the ability of the bacteria to remodel its outer mycobacterial membrane enabling enhanced accumulation of the EtBr due to a more permeable outer membrane. The reduction of MmpL3 activity in *Mtb* has been shown to alter the fluidity of the mycobacterial cell envelope and modify its permeability^65^. *Mtb* grown with controlled MmpL3 expression showed EtBr uptake like that of WT. However, when MmpL3 expression was down regulated, a 3-fold increase in EtBr uptake relative to the WT was observed. Similarly, as observed with the combinatory assays in this study, under-expression of MmpL3 resulted in hypersensitivity to drugs such as RIF, clarithromycin, erythromycin, and fidaxomicin, which are all large (>500 Da) and hydrophobic (log *P* > 2.5), which is indicative of modified cell wall permeability^65^.

The efflux assays demonstrated that the compounds marginally affected the efflux of EtBr in the absence of glucose, but no statistical significance was observed (Figure 4). Conversely, in the presence of glucose, Ad-1-ISO an Ad-2-ISO significantly increased the overall efflux of EtBr. The potential efflux transporters responsible for this activity fall into several superfamilies; ATP-Binding Cassette (ABC), Multidrug and Toxic Compound Extrusion (MATE), MFS, Resistance Nodulation and Cell Division (RND), and Small Multidrug Resistance (SMR) superfamily^66^. The identification of bacterial efflux pumps is still an area of ongoing research and it is clear that there is still a lot to elucidated^67^. One of the differences amongst these efflux mechanisms is their source of energy. Whilst the ABC family members transport substrates across membranes through the hydrolysis of ATP for energy^68^, other transporters including the MATE, MFS, RND, and SMR superfamilies utilise the proton-motive force. This proton-motive force is generated by H^+^ transfer or the electrochemical gradient of Na^+^ to supply energy^69^. Our data would therefore be suggestive of an increase in the permeability of the outer mycolic membrane as a result of reduced *de novo* synthesis of mycobacterial membrane components and not the inhibition of efflux or influx mechanisms.

### Radiolabel cell wall analysis 1D – FAMEs/MAMEs

**Figure 5.**
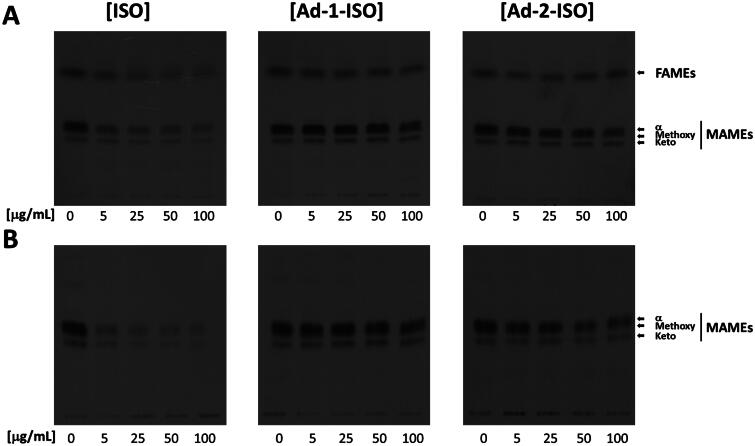
Representative TLC analyses of [1,2-^14^C]-labelled lipids newly synthesised in the presence of indicated concentrations of ISO, Ad-1-ISO and Ad-2-ISO extracted from *Mtb* mc^2^7902 pMV261. (a) Whole cell analysis; (b) Cell wall bound lipid analysis. Equal volumes of radioactivity were spotted for each sample. The developing solvent system comprised of chloroform-methanol (95:5). Fatty acid methyl esters (FAMEs), Mycolic acid methyl esters (MAMEs).

*Mtb* presents three aim types of mycolic acids within their cell wall: the di-cyclopropanated α-mycolic acids, and the oxygenated methoxy- and keto-mycolic acids. The synthesis of mycolic acids is essential for the survival of *Mtb in vivo*. To evaluate the effect of ISO and the two adamantyl hybrid compounds on mycolic acid biosynthesis, we extracted the mycolic acids and fatty acids as methyl esters from cultures grown in the presence of increasing concentrations of compound followed by labelling with [1,2-^14^C]-acetate. Cellular extracts were analysed by standardised TLC/autoradiography techniques. ISO activity and depletion of mycolate species present is in agreement with previous reports^15^^,^^16^^,^^18^^,^^45^, as ISO alters the synthesis of mycolates through inhibition of HadAB complex (Figure 5(a), left panel). ISO mycolate depletion was observed in both the cell wall-bound and whole cell analysis, indicative of primary synthesis inhibition and not transport or deposition. Depletion of mycolates into the cell wall was only observed at the highest concentrations tested of Ad-1-ISO whereas a more significant reduction in radiolabel incorporation was detected in the Ad-2-ISO samples (Figure 5(a), centre and right panel). This would suggest that the regioselectivity of the hybrid compounds has fostered a change in the intracellular target interaction, possibly to a non-mycolate synthetic protein, as the MICs indicate that both compounds are potent but have markedly different attributes in terms of mycolate synthesis inhibition. Notably, the adamantyl-1-urea containing AU1235 inhibits EphD, demonstrating that thiourea-based inhibitors have the potential to inhibit different enzymes within the cell^1^^,^^50^. Although non-essential *in vitro*, EphD has been shown to be essential for *Mtb* survival in macrophages^70^.

Contrary to the action of ISO in which inhibition of mycolic acid synthesis is observed in both the cell wall-bound and whole cell analysis indicative of predominantly primary synthesis inhibition. Whereas, Ad-2-ISO-mediated depletion was more readily observed in the bound cell wall analysis, suggesting a link to synthesis, transport, and deposition (Figure 5(b), right panels). To further investigate these mycolate profiles, the extracts were also run on silver TLC (Figure S1) to check possible changes to the saturation of the mycolic acids brought about by possible desaturation inhibition, however, no changes were observed in all samples tested.

### Radiolabel cell wall analysis 1D – 10:10:3 extraction ran with CHCl_3_/CH_3_OH/H_2_O (65:25:4, v/v/v)

**Figure 6.**
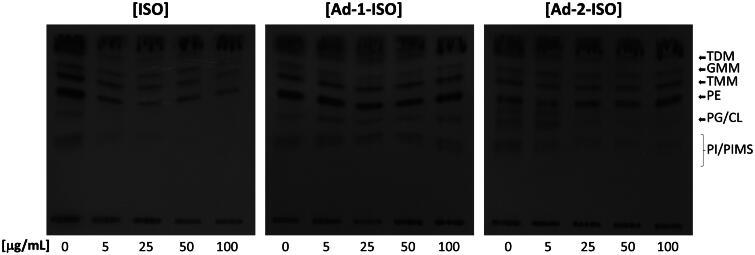
Representative TLC analyses of [1,2-^14^C]-labelled lipids newly synthesised in the presence of indicated concentrations of ISO, Ad-1-ISO and Ad-2-ISO extracted from *Mtb* mc^2^7902 pMV261. Equal volumes of the chloroform-methanol-water 10:10:3 extraction radioactivity was spotted for each sample. The developing solvent system comprised of chloroform-methanol-water (65:25:4). Trehalose dimycolate (TDM), Glucose monomycolate (GMM), Trehalose monomycolate (TMM), Phosphatidylethanolamine (PE), Phosphatidylglycerol/Cardiolipin (PG/CL), Phosphatidylinositol/Phosphatidylinositol mannosides (PI/PIMS).

Trehalose dimycolate (TDM) levels in the extractable lipid fraction gradually increase upon EMB treatment. This accumulation of mycolates in TDM was observed due to the loss of terminal hexa-arabinofuranoside attachment sites for mycolates^71^. In addition, SQ109 treatment results in the depletion of TDM through targeting of MmpL3. Previously, it was shown that despite the overall reduction in mycolic acids in both the cell wall skeleton and TDM, analysis of total fatty acids by saponification of whole cells showed a net accumulation of mycolic acid^3^. This accumulation was also shown to be due to the increase in the intensity of the spot corresponding to TMM upon TLC analysis of the whole cell fractions^3^. To investigate whether ISO and the two adamantyl hybrid compounds affect the synthesis of other mycobacterial lipids as with SQ109, addressing the potential of additional cell wall-associated targets, the total cellular lipids (and fractionated apolar lipids and polar phospholipids) were extracted and analysed following drug treatment. Firstly, a decrease in TDM and glucose monomycolate was observed with ISO and Ad-2-ISO, consistent with inhibition of mycolic acid biosynthesis (Figure 6). At the highest concentrations of Ad-1-ISO and Ad-2-ISO there was a slight increase in the TMM levels but was not observed with ISO. This is further indicative of Ad-2-ISO being linked to transport and deposition rather than synthesis. Secondly, no discernible differences were observed in lipid composition when apolar lipids and polar phospholipids were analysed by autoradiography-TLC (Figure S2-7).

## Discussion

In recent years, SQ109 has emerged as a promising drug against a variety of bacterial pathogens^5^^,^^72^. In addition to bacterial diseases, SQ109 is a potent drug against Chagas disease caused by *Trypanosoma cruzi*, *Plasmodium faciparum* and *Leishmania donovani*^73–75^ via disruption of the intracellular Ca^2+^ homeostasis along with collapsing the electrochemical potential of mitochondria. Whereas ISO has been demonstrated to show antimycobacterial specificity^11^. Utilising previous efficacious structures is one approach to developing novel compounds to aid in the fight against *Mtb*. Herein, we have generated two regioisomeric analogues of a hybrid SQ109-ISO compound with demonstrable activity against drug-sensitive and drug-resistant *Mtb* whilst also displaying limited mammalian toxicity *in vitro* when compared to other antitubercular agents.

In combinatory assays, a reduction in the MIC of SQ109 in the presence of Ad-1-ISO and Ad-2-ISO was observed, however, it was deemed that only Ad-2-ISO in combination with SQ109 demonstrated a significant synergy. We have attributed this to the structural similarity of the compounds and the possible dual targeting of MmpL3 as well as secondary targets. Overexpression of MmpL3 conferred an approximate 2-fold increase in resistance to Ad-2-ISO similar to the resistance observed with SQ109 treatment. The inhibitory effect of SQ109 (25 µM to 50 µM on solid media) was only partially reversed in the *M. smegmatis* strains overexpressing MmpL3^46^. Interestingly, in *M. abscessus,* high levels of resistance to MmpL3 inhibitors was only observed when the a mutant allele of *mmpL3* (Ala309➔Pro) was overexpressed, whereas the WT allele did not confer significant resistance^76^. Therefore, our observations suggest the Ad-2-ISO hybrid has a similar mode of action to SQ109. Additionally, SQ109 has demonstrated a reduction in TDM and glucose monomycolate within the cell wall skeleton, similar to what was seen with Ad-2-ISO, which aligns with the inhibition of mycolic acid biosynthesis through MmpL3^3^. At the highest concentration tested, as with SQ109, there was a significant increase in the TMM levels upon treatment with Ad-2-ISO. In addition, *M. smegmatis* mc^2^155 demonstrated a 2-fold increase in the resistance to AU1235 only upon expression of the mutated version of MmpL3-*Mtb*-G253E in a *mmpL3* knockout strain, whereas the overexpression of the wild-type version of MmpL3-*Mtb* did not confer resistance. Notwithstanding this, the overexpression of wild-type MmpL3-*Mtb* induced hypersensitisation of the organism to AU1235^2^. This highlighting the significance of the resistance phenotype observed to the Ad-2-ISO upon MmpL3 overexpression.

The combination assays for SQ109 and Ad-1-ISO did not result in a significant synergy between the compounds, which when evaluated alongside the MIC data and TLC analysis, is suggestive that this compound is interacting differently with the bacteria suggestive of a potentially different target. The overexpression of MmpL3, or any other gene tested in this study, did not confer any discernible resistance to Ad-1-ISO. These results suggest that the change in regiochemistry of the adamantyl is responsible for this potential change in cellular targets. Ad-1-ISO is closer in structural relationship to AU1235 which has been proposed to target the epoxide hydrolase, EphD^50^^,^^51^, and MmpL3^2^, however, the possibility that EphD is the target would need significant further work to prove or disprove otherwise.

**Figure 7.**
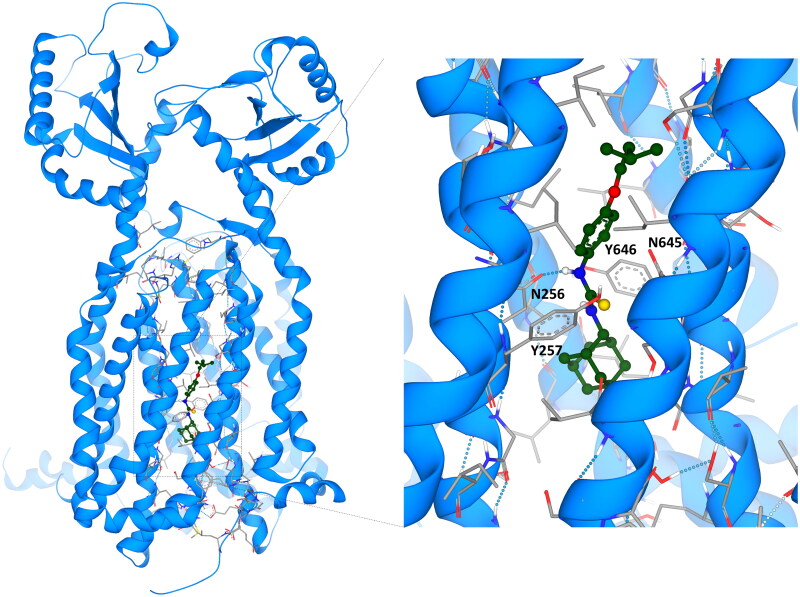
SeeSAR modelling of the MmpL3 holo-form with Ad-2-ISO (PDB ID: 6AJG).

To further demonstrate potential mode of action of our hybrid compounds we conducted modelling analysis using SeeSAR and the MmpL3 inhibitor-bound holo-form with SQ109 (PDB ID: 6AJG)^46^. This analysis afforded predictive binding affinities of the ISO, SQ109 and hybrid compounds (Figure 7). No water molecules were observed in the original structures as it has been shown that the channel opens to enable proton translocation via a water wire^77^. Interestingly, we were unable to generate models of the Ad-1-ISO into the binding channel of SQ109. Examination of the predicted 3-dimensional structure of the two hybrid compounds showed that Ad-2-ISO is relatively linear, as per SQ109, whereas Ad-1-ISO is kinked in nature. This may explain the reduced synergy between these compounds in the MIC analysis and is further supportive of a different target for this stereoisomer. The predictive modelling of Ad-2-ISO demonstrated a high level of estimated affinity (0.00045 nM) for MmpL3, which pleasingly supports our biological analysis but further protein interaction studies will be required to validate this observation (Table S5). The predicted binding orientation has a similar conformation to SQ109 (Figure 7), however, in SQ109 hydrogen bonding occurs between the secondary amine closest to the adamantyl moiety and the binding channel Asp645 whereas our Ad-2-ISO thiourea is predicted to interact with the Asp256 carbonyl via the NH-Aniline (Table S5)^77^. As with SQ109 the adamantyl moiety of Ad-2-ISO locates in a V-shaped pocket formed by Phe260 and Phe649 at the base of the water wire channel^46^. Analysis of the transmembrane domain (TMD) pairs showed that Asp256–Tyr646 and Tyr257–Asp645 are in close interaction, the Phe260 and Phe649 bottleneck is at 0 Å, effectively closing the channel. As these residues are highly conserved among mycobacterial sequences, the importance of this predicted interaction of Ad-2-ISO with MmpL3 cannot be overstated.

**Figure 8.**
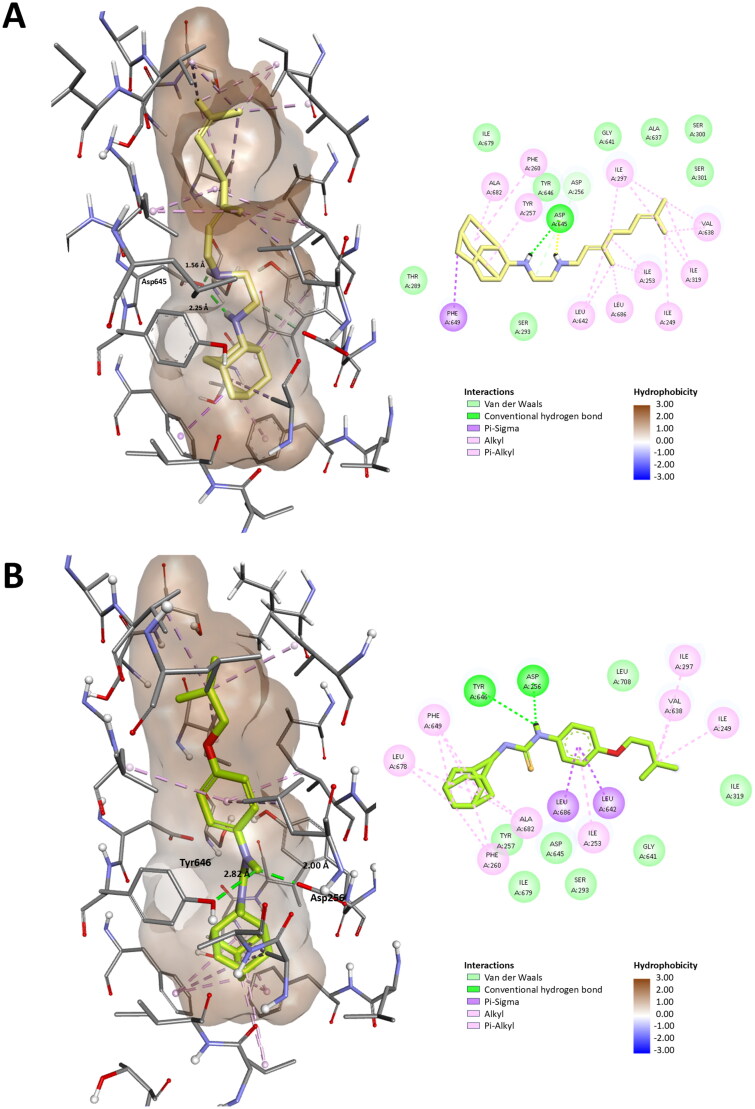
The binding orientation and binding interactions of Ad-2-ISO with the SQ109 binding channel of MmpL3 compared to the co-crystallised ligand (PDB ID: 6AJG). A) SQ109 remodelled B) Ad-2-ISO. Right panel shows the 2D interaction maps of the two compounds. Interactions are colour coded as per the inserted key.

Protonation of the Asp645 was deemed to induce the conformational changes that will open the bottleneck and allow for proton transport through the TMD channel and is shown to interact with the thiourea NH closest to the adamantyl unit in SQ109. If our Ad-2-ISO binds to the other Asp-Tyr pair, as per the prediction, it may retain activity in SQ109 resistant mutants. However, several residues within the transmembrane segments of MmpL3 are associated with SQ109 resistance in *Mtb* with Tyr252 (Tyr257 in *M. smeg*), Phe255 (Phe260 in *M. smeg*), Phe644 (Phe649 in *M. smeg*) being prominent. Therefore, it is possible that Ad-2-ISO would retain activity against a subset of these SQ109 mutants but it is likely that cross resistance will be observed. The binding orientation and binding interactions of Ad-2-ISO with the SQ109 binding channel of MmpL3 compared to the co-crystallised ligand (PDB ID: 6AJG), demonstrated that like SQ109, the Ad-2-ISO variant occupied the whole channel and 2D analysis revealed similar compound-protein interactions (Figure 8). As with AU1235, the results strongly supported the assumption that MmpL3 serves as a target for Ad-2-ISO, however the very nature of the protein also makes it possible that it acts as an efflux pump for the compound^2^.

**Figure 9.**
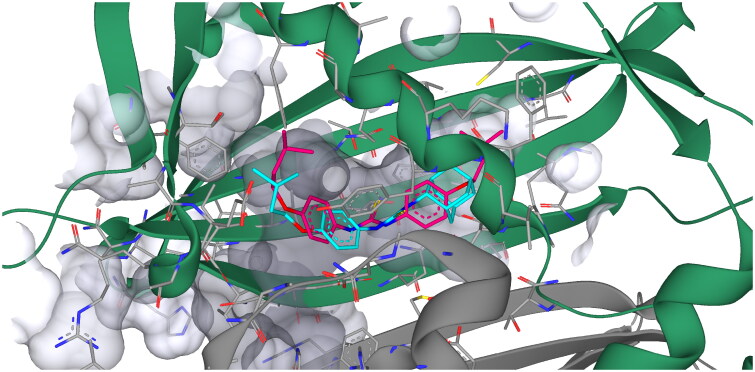
SeeSAR modelling of HadAB (PDB ID: 4RLW) with ISO and Ad-2-ISO. ISO – magenta, Ad-2-ISO cyan.

As a final modelling experiment the hybrid compounds modelled using SeeSAR and the HadAB-complex crystal structure (PDB ID: 4RLW) and the HadBC-complex from *Mtb* (PDB ID: 5ZY8) due to the resistance observed against Ad-2-ISO upon overexpression of HadABC. As previously observed, ISO bound to HadAB but not to the HadBC complex^45^. The Ad-2-ISO compound bound with similar predicted affinity to that of ISO to the HadAB complex (20.41 nM and 30.92 nM for Ad-2-ISO and ISO, respectively) and in a similar binding orientation (Figure 9). This modelling is supportive of Ad-2-ISO potentially hitting multiple targets within the *Mtb* cell. The MIC data, overexpression studies and radiochemical labelling further support the possible inhibition of the FAS-II cycle of *Mtb* via HadAB. The Ad-1-ISO model against both complexes again provided no plausible dockings which would afford a predictive binding. Taken in conjunction, the modelling results are further indicative of a potential difference in the mode of action of these regioisomeric compounds.

## Conclusion

In conclusion, our genetic and biochemical studies suggest that these structurally similar pharmacophores bind to different proteins within *Mtb*, highlighting the need for careful consideration when producing regioisomeric species. Furthermore, this study demonstrates that utilisation of previous efficacious structures is a valid approach to developing novel series of compound to generate promising drugs against *Mtb*.

## Supplementary Material

JEIMC_Dr_AK_Brown_Supplemental_ Clean.docx

## Data Availability

The main text and the Supplementary Material contain all the necessary data to evaluate this study’s conclusions. Additional data generated during the current study are available from the corresponding author upon reasonable request.
