# Supplementary material for: Structural isomerisation affects the antitubercular activity of adamantyl-isoxyl adducts
Source: J Enzyme Inhib Med Chem. 2025 May 21;40(1):2502600. doi: 10.1080/14756366.2025.2502600 (PMC12096669; doi:10.1080/14756366.2025.2502600)
Supplement: JEIMC_Dr_AK_Brown_Supplemental_ Clean.docx [file IENZ_A_2502600_SM4178.docx]

Supplemental

Structural isomerisation affects the antitubercular activity of adamantyl-isoxyl adducts.

Yucheng Lu^1^, Daniel Partleton^1^, Filibus M. Gugu^1,2^, Ahmed Y. G. Alhejaili^1^, Samuel Norris^3^, J. Jonathan Harburn^3^, Jason H. Gill^4,5^, Jonathan D. Sellars^1,4^, and Alistair K. Brown^1*^

^1^Biosciences Institute, Faculty of Medical Sciences, Newcastle University, Newcastle upon Tyne, NE2 4HH, UK

^2^Department of Microbiology, Plateau State University Bokkos, P.M.B. 2012. Jos, Nigeria

^3^Chemistry, School of Natural and Environmental Sciences, Newcastle University, Newcastle upon Tyne, NE1 7RU, UK

^4^School of Pharmacy, Faculty of Medical Sciences, King George VI Building, Newcastle upon Tyne, NE1 7RU, UK

^5^Translational and Clinical Research Institute, Faculty of Medical Sciences, Newcastle University, Newcastle upon Tyne, NE2 4HH, UK

*Corresponding author Alistair K. Brown ([alistair.brown2@ncl.ac.uk](mailto:alistair.brown2@ncl.ac.uk))

**Supplementary Biological Procedures**

**Plasmid construction and *Mycobacterium* manipulation**

PCR products corresponding to the correct molecular weight of the genes of interest were generated using standard protocols for Vent DNA polymerase (New England Biolabs), gene specific primers, and *Mtb* H37Rv gDNA (Sup. Table 1). PCR fragments for the genes of interest were cloned into the *Bam*HI *Hind*III sites of pMV261 to generate the plasmids used in this study (Sup. Table 2). All new plasmids generated were validated by sanger sequencing (Eurofins Genomics).

**Sup. Table 1** – PCR primers used in this study.

**pMV261-*desA3*_For** AAAAAAGGATCCAGCGATCACTGACGTCGAC

**pMV261-*desA3*_Rev** AAAAGAAAGCTTTTAGGCTGCCAGATCGTCG

**pMV261-*ethA*_For** AAAAAAGGATCCAACCGAGCACCTCGACGTTGTCATC

**pMV261-*ethA*_Rev** AAAAAAAAGCTTCTAAACCCCCACCGGGGCA

**pMV261-*mmpL3*_For** AAAAAGAGATCTATTCGCCTGGTGGGGTCG

**pMV261-*mmpL3*_Rev** AAAAAAAAGCTTTTAAAGGCGTCCTTCGCGGC

**pMV261-*pks13*_For** AAAAAAGGATCCAGCTGACGTAGCGGAATCC

**pMV261-*pks13*_Rev** AAAGGAAAGCTTTCACTGCTTGCCTACCTCA

**Sup. Table 2** – Plasmids and *Mycobacterium* strains used in this study.

| **Plasmid and Strain** | **Description** | **Source or reference** |
| --- | --- | --- |
| **Plasmids** |  |  |
| pMV261 | *E. coli*-mycobcaterial shuttle plasmid, *hsp60* promoter, Kan^R^ | Stover *et al*., 1991[1] |
| pMV261-*hadABC* | *hadABC* cloned in pMV261, Kan^R^ | Abrahams *et al*., 2016[2] |
| pMV261-*desA3* | *desA3* cloned in pMV261, Kan^R^ | This study |
| pMV261-*ethA* | *ethA* cloned in pMV261, Kan^R^ | This study |
| pMV261-*mmpL3* | *mmpL3* cloned in pMV261, Kan^R^ | This study |
| pMV261-*pks13* | *pks13* cloned in pMV261, Kan^R^ | This study |
|  |  |  |
| **Strains** |  |  |
| *Mtb* mc^2^7902 | *ΔleuCD ΔpanCD ΔargB,* Leucine, pantothenate and arginine triple auxotroph | Vilcheze *et al*., 2018[3] |
| *Mtb* mc^2^8245 (INH^R^) | mc^2^7902 derived Δ2116169–2162530; Δ2116169–2162530 genome deletion, INH^R^ | Vilcheze *et al*., 2018[3] |
| *Mtb* mc^2^8247 (RIF^R^) | mc^2^7902 derived, *rpoB* (H445Y); *rpoB* His445 → Lys, RIF^R^ | Vilcheze *et al*., 2018[3] |
| *Mtb* mc^2^8250 (RIF^R^ & INH^R^) | mc^2^8247 derived, *rpoB* (H445Y) Δ2122397–2170320; *rpoB* His445 → Lys, RIF^R^, Δ2122397–2170320 genome deletion, INH^R^ | Vilcheze *et al*., 2018[3] |
| *Mtb* mc^2^8258 (RIF^R^ & INH^R^) | mc^2^8247 derived, *rpoB* (H445Y) *katG* (W438R); *rpoB* His445 → Lys, RIF^R^, *katG* Trp438 → Arg, INH^R^ | Vilcheze *et al*., 2018[3] |
| *Mtb* mc^2^7902 pMV261 | *Mtb* mc^2^7902 containing pMV261, Kan^R^ | This study |
| *Mtb* mc^2^8245 pMV261 | *Mtb* mc^2^8245 containing pMV261, Kan^R^ | This study |
| *Mtb* mc^2^8247 pMV261 | *Mtb* mc^2^8247 containing pMV261, Kan^R^ | This study |
| *Mtb* mc^2^8250 pMV261 | *Mtb* mc^2^8250 containing pMV261, Kan^R^ | This study |
| *Mtb* mc^2^8258 pMV261 | *Mtb* mc^2^8258 containing pMV261, Kan^R^ | This study |
| *Mtb* mc^2^7902-*hadABC* | *Mtb* mc^2^7902 containing pMV261-*hadABC*, Kan^R^ | This study |
| *Mtb* mc^2^7902-*desA3* | *Mtb* mc^2^7902 containing pMV261-*desA3*, Kan^R^ | This study |
| *Mtb* mc^2^7902-*ethA* | *Mtb* mc^2^7902 containing pMV261-*ethhA*, Kan^R^ | This study |
| *Mtb* mc^2^7902-*mmpL3* | *Mtb* mc^2^7902 containing pMV261-*mmpL3*, Kan^R^ | This study |
| *Mtb* mc^2^7902-*pks13* | *Mtb* mc^2^7902 containing pMV261-*pks13*, Kan^R^ | This study |

**Sup. Table 3** – Non-mycobacterial strains used in this study and growth media used.

| **Strain** | **Media used for MIC determination** |
| --- | --- |
| *Bacillus cereus* NCTC 7464 | Mueller Hinton broth |
| *Streptococcus pyogenes* NCTC 8198 | Mueller Hinton broth + 1% Lysed Horse Blood |
| *Streptococcus* *agalactiae* NCTC 8181 | Mueller Hinton broth + 1% Lysed Horse Blood |
| *Enterococcus faecalis* NCTC 775 | Mueller Hinton broth |
| *Enterococcus* *faecium* NCTC 7171 | Mueller Hinton broth |
| *Staphylococcus aureus* NCTC 2981 | Mueller Hinton broth |
| *Klebsiella pneumoniae* NCTC 9633 | Mueller Hinton broth |
| *Acinetobacter baumannii* NCTC 12156 | Mueller Hinton broth |
| *Pseudomonas aeruginosa* DSM 19880 | Mueller Hinton broth |
| *Enterobacter cloacae* NCTC 10005 | Mueller Hinton broth |
| *Salmonella enterica* NCTC 6754 | Mueller Hinton broth |
| *Escherichia coli* K12 | Mueller Hinton broth |

**Sup. Table 4** – Non-mycobacterial REMA assay MIC determination and Swiss ADME prediction.

|  | |  |  | | **Minimum inhibitory concentration 95% (μg/mL)** | | | | | | | | | | |  |  |  |  |  |  |  |
| --- | --- | --- | --- | --- | --- | --- | --- | --- | --- | --- | --- | --- | --- | --- | --- | --- | --- | --- | --- | --- | --- | --- |
|  |  | **ISO** | | **Ad-1-ISO** | | **Ad-2-ISO** | **RIF** | **INH** | **LZD** | **SQ109** | **ETH** | | **EMB** | |  |  |  |  |  |  |  |  |
|  | *B. cereus* NCTC 7464 | >32 | | >32 | | >32 | 0.045 | >32 | 1.755 | >32 | >32 | | >32 | |  |  |  |  |  |  |  |  |
|  | *S. pyogenes* NCTC 8198 | >32 | | >32 | | >32 | 6.159 | >32 | 0.543 | >32 | >32 | | >32 | |  |  |  |  |  |  |  |  |
|  | *S. agalactiae* NCTC 8181 | >32 | | >32 | | >32 | 0.080 | >32 | 2.101 | >32 | >32 | | >32 | |  |  |  |  |  |  |  |  |
|  | *E. faecalis* NCTC 775 | >32 | | >32 | | >32 | >12.8 | >32 | 0.921 | 21.08 | >32 | | >32 | |  |  |  |  |  |  |  |  |
|  | *E. faecium* NCTC 7171 | >32 | | >32 | | >32 | 1.733 | >32 | 0.158 | 20.1 | >32 | | >32 | |  |  |  |  |  |  |  |  |
|  | *S. aureus* NCTC 2981 | >32 | | >32 | | >32 | <0.0125 | >32 | 23.72 | 16.87 | >32 | | >32 | |  |  |  |  |  |  |  |  |
|  | *K. pneumoniae* NCTC 9633 | >32 | | >32 | | >32 | >12.8 | >32 | >32 | >32 | >32 | | >32 | |  |  |  |  |  |  |  |  |
|  | *A. baumannii* NCTC 12156 | >32 | | >32 | | >32 | 1.363 | >32 | 31.06 | 20.72 | >32 | | >32 | |  |  |  |  |  |  |  |  |
|  | *P. aeruginosa* DSM 19880 | >32 | | >32 | | >32 | >12.8 | >32 | >32 | >32 | >32 | | >32 | |  |  |  |  |  |  |  |  |
|  | *E. cloacae* NCTC 10005 | >32 | | >32 | | >32 | >12.8 | >32 | >32 | 16.83 | >32 | | >32 | |  |  |  |  |  |  |  |  |
|  | *S. enterica* NCTC 6754 | >32 | | >32 | | >32 | 12.76 | >32 | >32 | >32 | >32 | | >32 | |  |  |  |  |  |  |  |  |
|  | *E. coli* K12 | >32 | | >32 | | >32 | 3.034 | >32 | >32 | 17.35 | >32 | | >32 | |  |  |  |  |  |  |  |  |
| **Predicted Water Solubility** | |  |  | |  | | | | | | | | | | |  |  |  |  |  |  |  |
|  | iLOGP | 4.86 | | 4.37 | | 4.45 | 5.25 | 0.03 | 2.58 | 4.91 | 1.58 | | 2.47 | |  |  |  |  |  |  |  |  |
|  | ESOL Log S | -5.88 | | -5.48 | | -5.3 | -8.18 | -0.56 | -2.22 | -4.51 | -1.82 | | -0.46 | |  |  |  |  |  |  |  |  |
|  | ESOL Solubility (mg/mL) | 5.27E-04 | | 1.22E-03 | | 1.86E-03 | 5.47E-06 | 3.77E+01 | 2.03e+00 | 1.01E-02 | | 2.54E+00 | | 7.05E+01 | | |  |  |  |  |  |  |
|  | ESOL Solubility (mol/L) | 1.32E-06 | | 3.28E-06 | | 4.99E-06 | 6.65E-09 | 2.75E-01 | 6.01e-03 | 3.06E-05 | | 1.53E-02 | | 3.45E-01 | | |  |  |  |  |  |  |
|  | ESOL Class | Moderately soluble | | Moderately soluble | | Moderately soluble | Poorly soluble | Very soluble | Soluble | Moderately soluble | Very soluble | | Very soluble | |  |  |  |  |  |  |  |  |
| **Predicted Pharmacokinetics** | |  |  | |  | | | | | | | | | | |  | |  |  |  |  |  |
|  | GI absorption | High | | High | | High | Low | High | High | High | High | | High | |  |  |  |  |  |  |  |  |
|  | BBB permeant | No | | No | | Yes | No | No | No | Yes | No | | No | |  |  |  |  |  |  |  |  |
|  | Pgp substrate | No | | Yes | | No | Yes | No | Yes | No | No | | No | |  |  |  |  |  |  |  |  |
|  | CYP1A2 inhibitor | Yes | | No | | No | No | No | No | No | No | | No | |  |  |  |  |  |  |  |  |
|  | CYP2C19 inhibitor | Yes | | Yes | | Yes | No | No | No | No | No | | No | |  |  |  |  |  |  |  |  |
|  | CYP2C9 inhibitor | Yes | | Yes | | Yes | No | No | No | No | No | | No | |  |  |  |  |  |  |  |  |
|  | CYP2D6 inhibitor | Yes | | Yes | | Yes | No | No | No | Yes | No | | No | |  |  |  |  |  |  |  |  |
|  | CYP3A4 inhibitor | Yes | | No | | No | No | No | No | No | No | | No | |  |  |  |  |  |  |  |  |
|  | log Kp (cm/s) | -4.2 | | -4.41 | | -4.62 | -7.44 | -7.63 | -7.87 | -4.69 | -6.55 | | -7.6 | |  |  |  |  |  |  |  |  |
| **Druglikeness** | |  |  | |  | | | | | | | | | | |  | |  |  |  |  |  |
|  | Lipinski #violations | 1 | | 0 | | 0 | 3 | 0 | 0 | 1 | 0 | | 0 | |  |  |  |  |  |  |  |  |
|  | Ghose #violations | 1 | | 0 | | 0 | 3 | 3 | 0 | 0 | 0 | | 0 | |  |  |  |  |  |  |  |  |
|  | Veber #violations | 1 | | 0 | | 0 | 1 | 0 | 0 | 0 | 0 | | 0 | |  |  |  |  |  |  |  |  |
|  | Egan #violations | 1 | | 0 | | 0 | 1 | 0 | 0 | 0 | 0 | | 0 | |  |  |  |  |  |  |  |  |
|  | Muegge #violations | 1 | | 1 | | 1 | 5 | 1 | 0 | 1 | 1 | | 0 | |  |  |  |  |  |  |  |  |
|  | Bioavailability Score | 0.55 | | 0.55 | | 0.55 | 0.17 | 0.55 | 0.55 | 0.55 | 0.55 | | 0.55 | |  |  |  |  |  |  |  |  |

**Figure S1 – Cell wall bound mycolates 2D Silver-TLC**


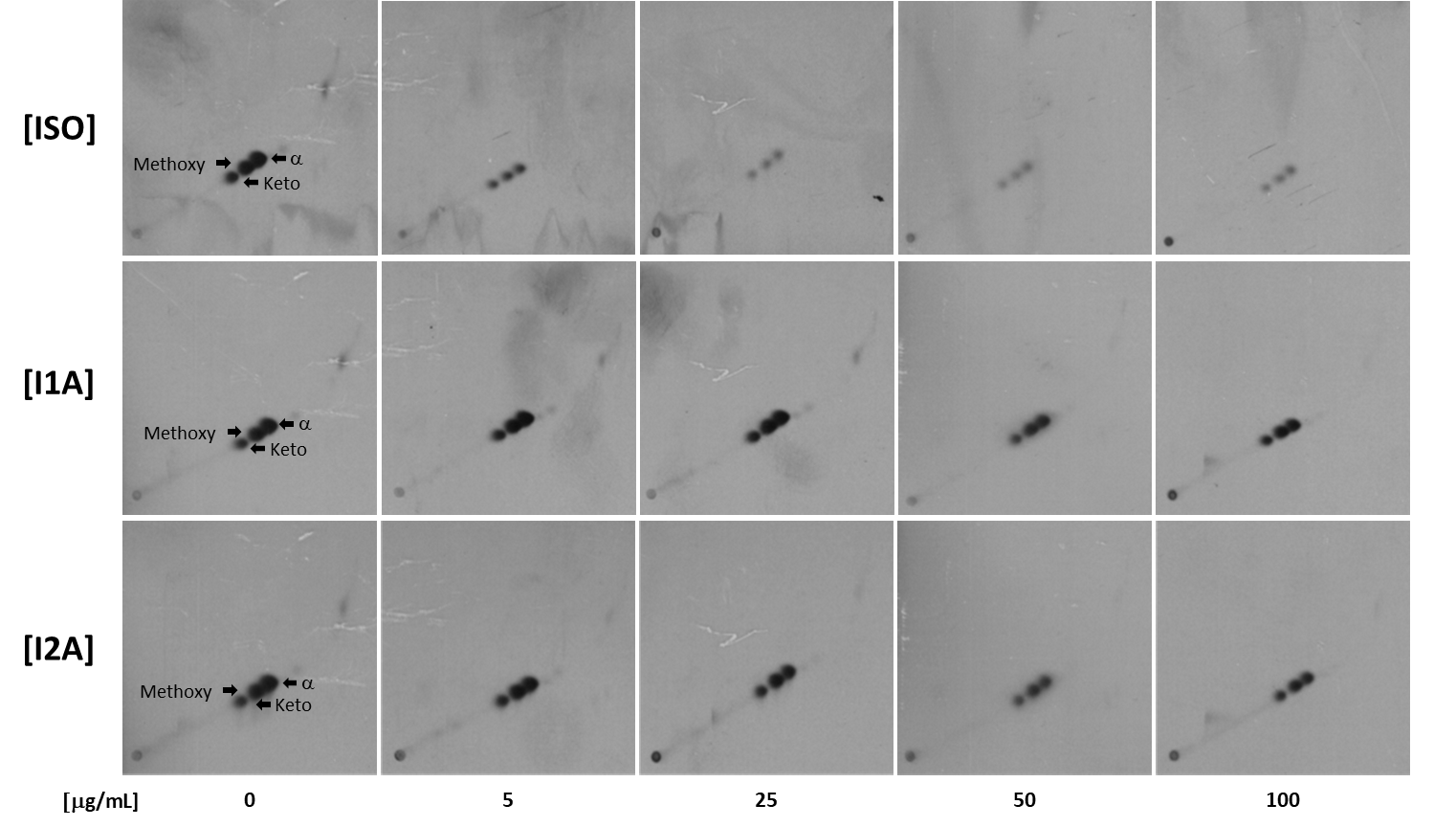


**Polar/Apolar Lipid Extraction and analysis**

Radiolabelled cell pellets were resuspended in 2 mL of chloroform/methanol/H_2_O (10:10:3) and incubated at 50°C for 3 hours. Cell debris were pelleted by centrifugation (3,000 x *g* 10 min) and supernatant was carefully transferred into a new glass tube. This step was repeated to yield a total of 4 ml of supernatants. At this stage, the liquid contained polar and apolar lipids, and the cell debris contained cell-wall bound mycolic acids.

1.75 mL of chloroform and 0.75 mL of water were added to the liquid mixture, which was then mixed thoroughly on a rotator at 15 rpm for 30 minutes. The upper aqueous phase and the intermediate phase were removed with glass pipette and discarded. 2 mL of chloroform/methanol/H_2_O (3:47:48) was added to the lower organic phase and the sample was mixed on rotor for 10 minutes. After centrifuge at 1000 x g for 3 minutes, upper and intermediate phases were discarded. The chloroform/methanol/H_2_O (3:47:48) wash was repeated for two more times to clean up the organic phase containing the lipids, which was then dried on a heating block at 50°C under stream of air.

Lipids and MAMEs were dissolved in 200 µL of chloroform/methanol (2:1) or DCM. 5 µL of Lipids/MAMEs were added to 5 mL of scintillation fluid (Ecoscint A) inside of scintillation bottle and the radioactivity of the sample was measured in CPM using a Beckman Coulter LS 6500 Multi-Purpose Scintillation Counter. 50,000 CPM of lipid/MAMEs sample were spotted across a 1 cm line on a 10 cm x 10 cm silica gel for one dimensional TLC or in a 0.4 cm x 0.4 cm spot on a 6.7 cm x 6.7 cm silica gel for two-dimensional TLC. The dried TLC plates were placed into a cassette along with an X-ray film (Scientific Laboratory Supplies). Exposure can take from 1 to 6 days depending on the amount of radioactivity placed onto the silica plates. After exposure, the films were submerged in developing solution (Tetenal) for 5 minutes, rinsed with water, and submerged in fixing solution (Tetenal) and gently shaken for another 5 minutes. After being rinsed and dried, the films would allow for visualisation of the lipid/MAMEs profile of *Mtb* samples.

| TLC System | Direction 1 | Direction 2 |
| --- | --- | --- |
| A | Petroleum ether/ethyl acetate (98:2) x3 | Petroleum ether/acetone (98:2) x1 |
| B | Petroleum ether/acetone (95:5) x3 | Toluene/acetone (95:5) x1 |
| C | Chloroform/methanol (96:4) x1 | Toluene/acetone (80:20) x1 |
| D | Chloroform/methanol/water (100:14:0.8) x1 | Chloroform/acetone/methanol/water (50:60:2.5:3) x1 |
| E | Chloroform/methanol/water (60:30:6) x1 | Chloroform/acetic acid/methanol/water (40:25:3:6) x1 |

**Figure S2 – Apolar Lipids System A**


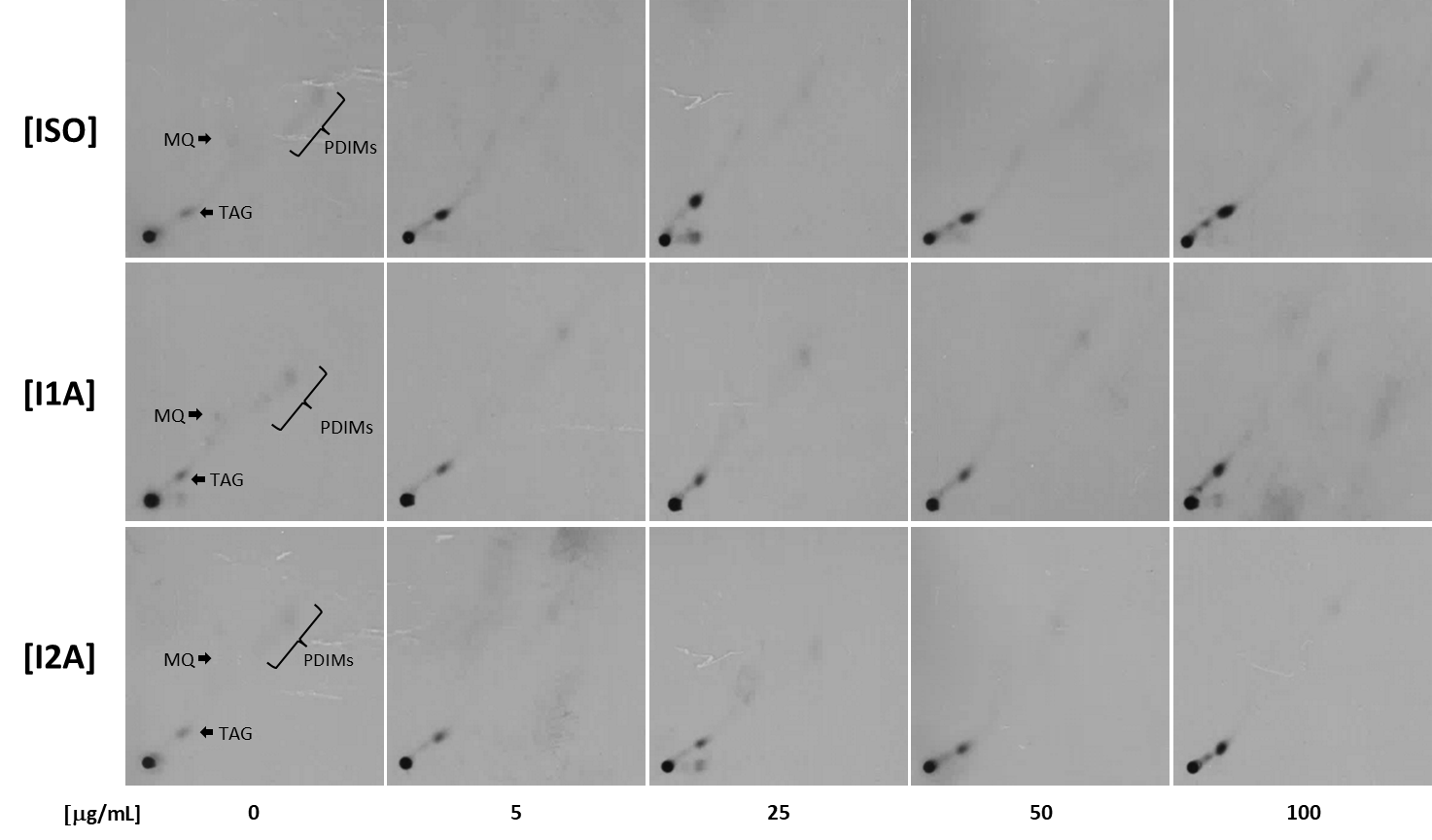


**Figure S3 – Apolar Lipids System B**


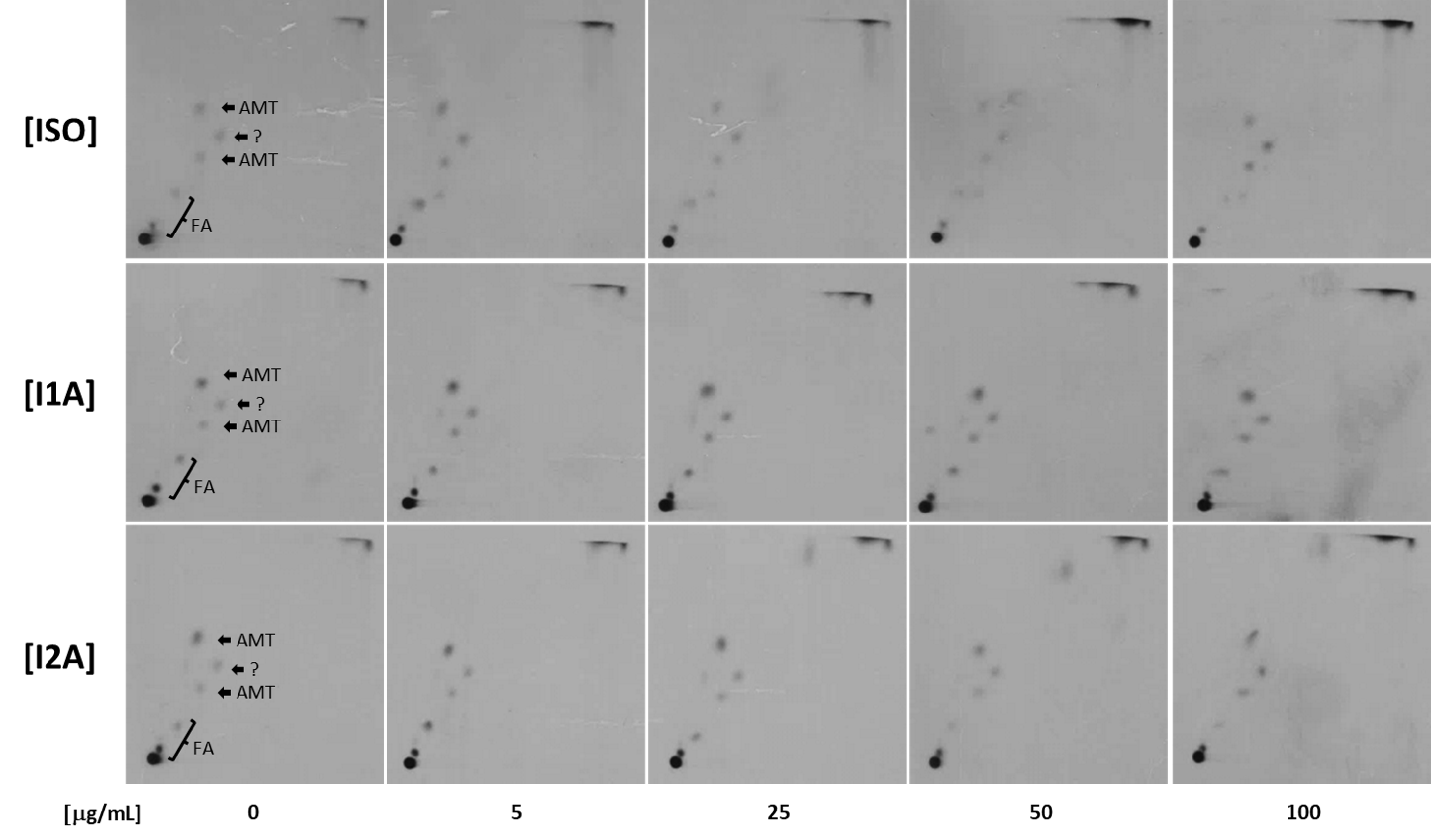


**Figure S4 – Apolar Lipids System C**

**
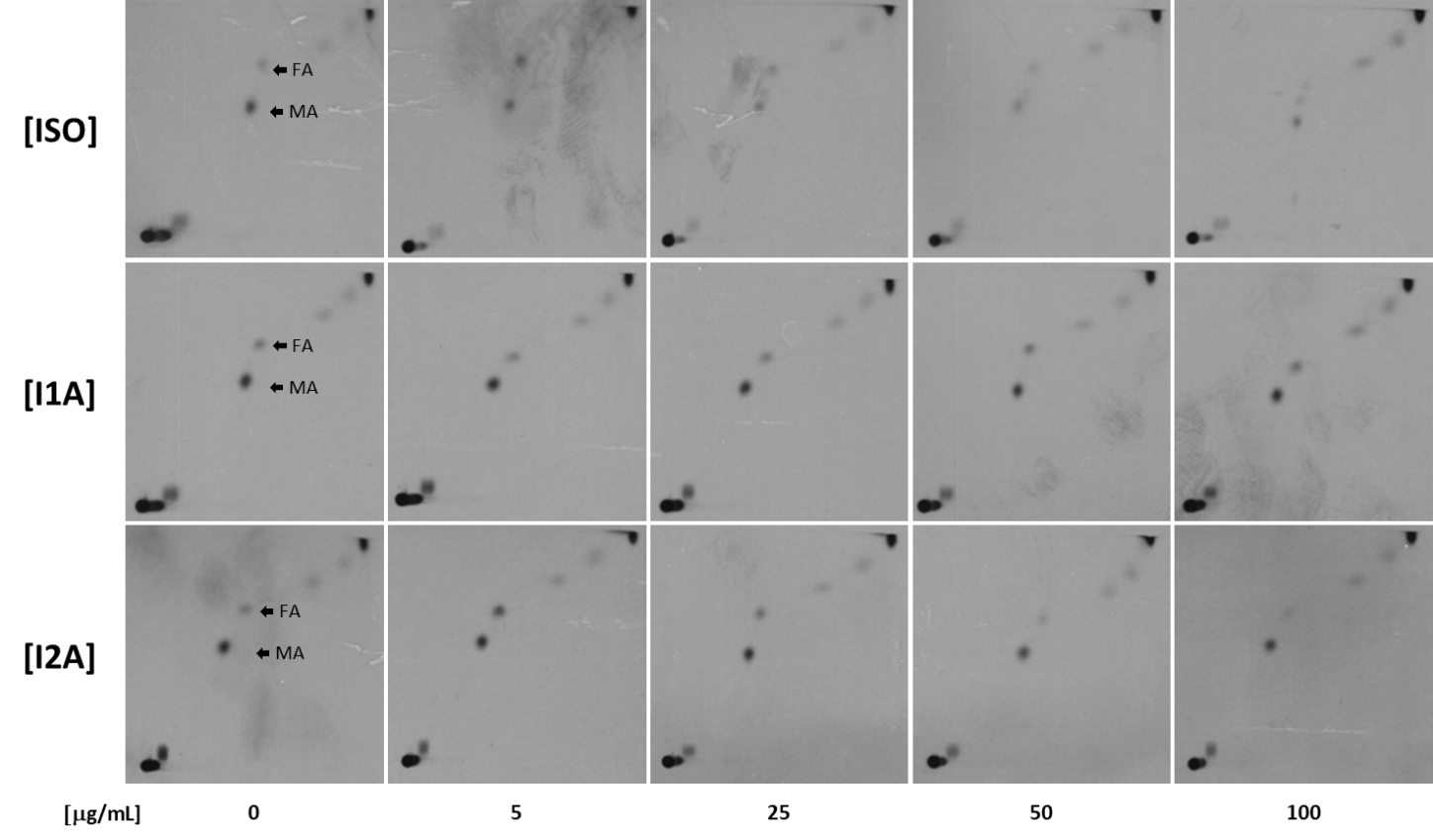
**

**Figure S5 – Apolar Lipids System D1**

**
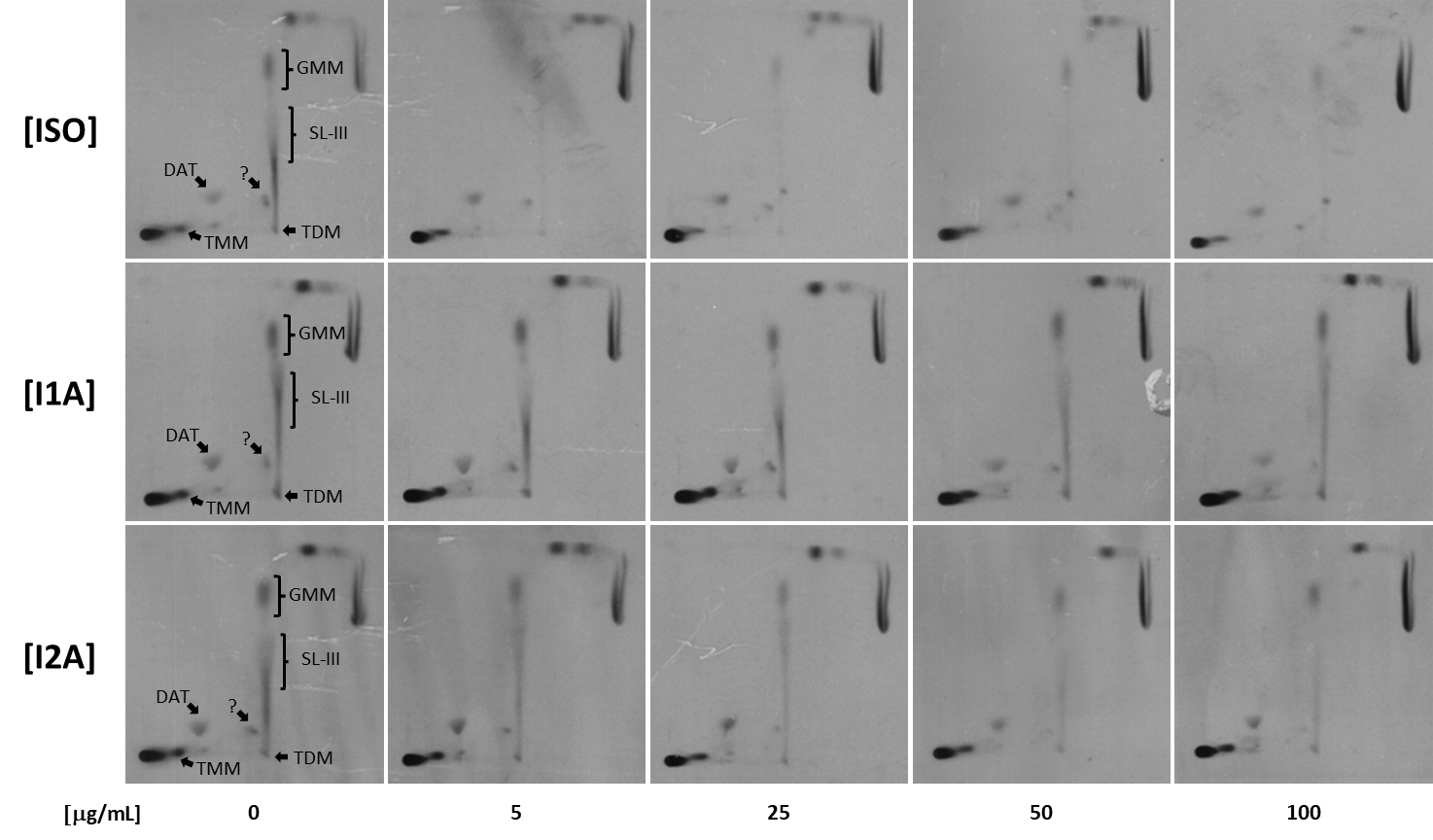
**

**Figure S6 – Polar Lipids System D2**


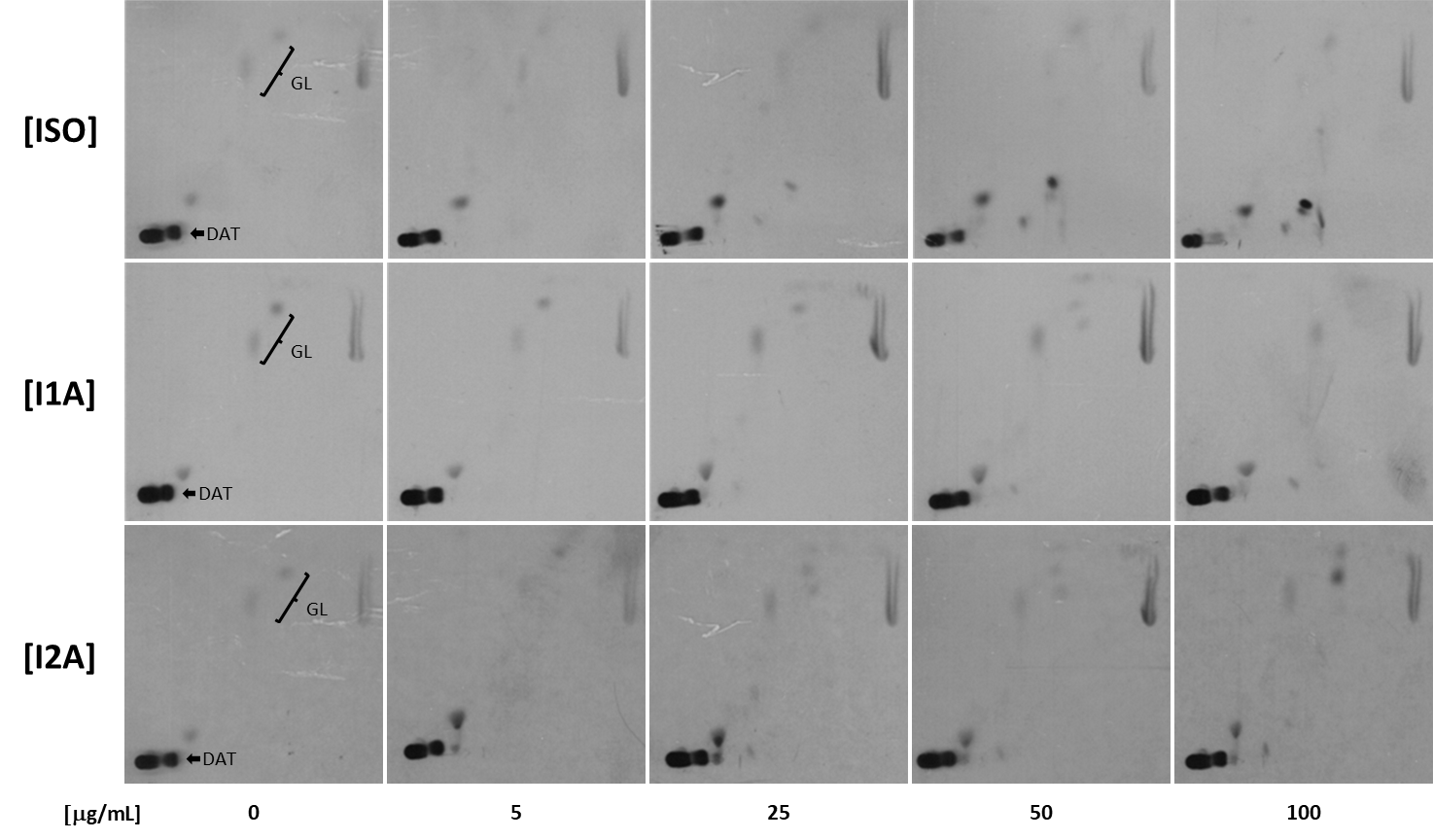


**Figure S7 – Polar Lipids System E**


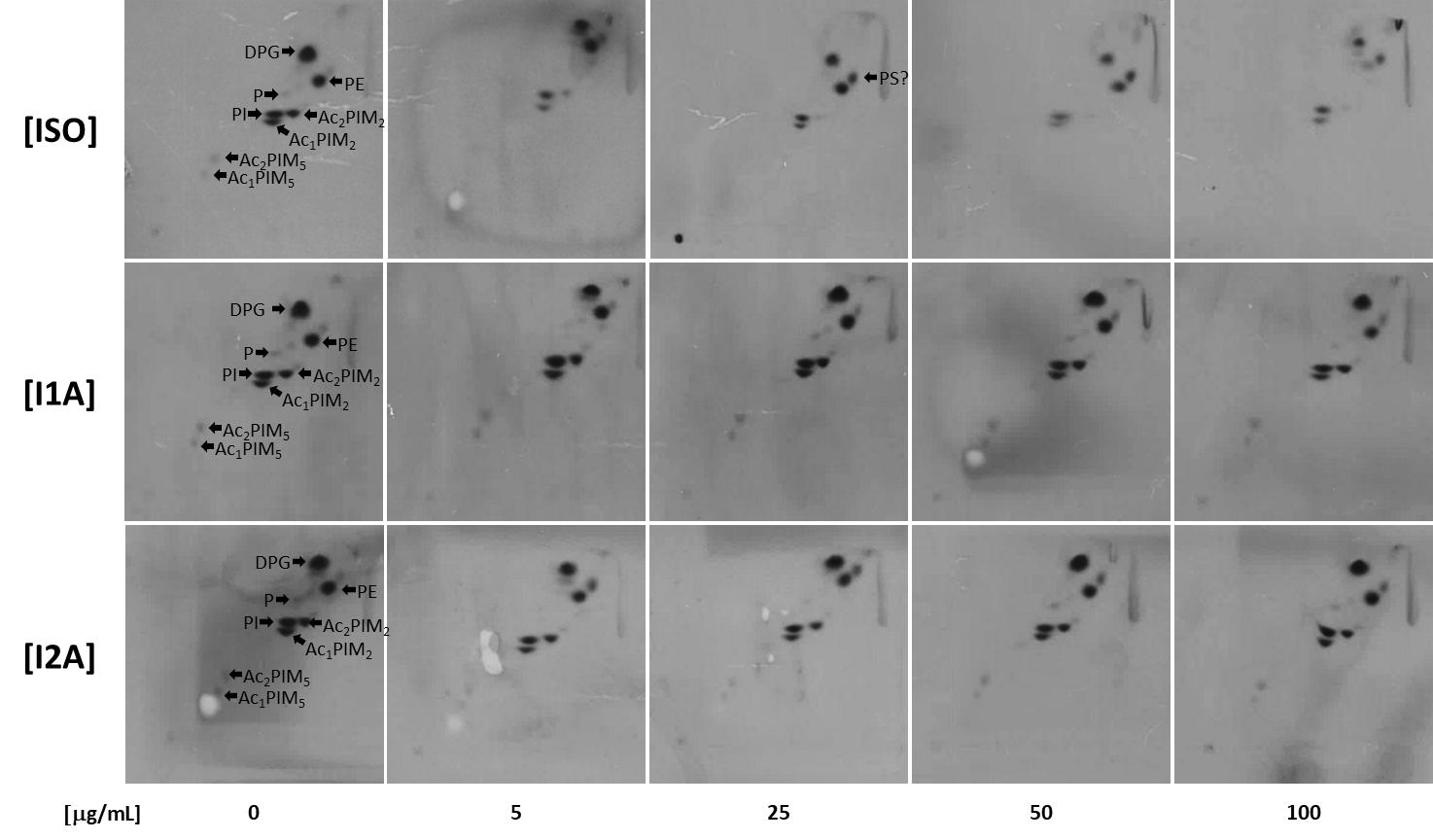


**Table S5** Modelling results against selected essential or fatty acid related mycobacterial drug targets.[4]

|  |  |  |  | **Predicted affinity (nM)^a^** | |  |  |  |
| --- | --- | --- | --- | --- | --- | --- | --- | --- |
| **Rv No.** | ***Mtb* Protein target** | **Targeted pathway** | **PDB ID** | **Ad-1-ISO** | **Ad-2-ISO** | **Predicted residue interaction, compound component** | **Dist. Å, atom** | **References** |
| Rv3790 | Decaprenylphosphoryl-ß-d-ribofuranose oxidoreductase (DprE1)^b^ | Arabinogalactan biosynthesis | 4P8C | - | - |  |  | [5] |
| Rv0533c | ß-ketoacyl acyl carrier protein synthase (FabH)^b^ | Mycolic acid biosynthesis | 1M1M | - | - |  |  | [6] |
| Rv2245 | ß-ketoacyl acyl carrier protein synthase (KasA)^b^ | Mycolic acid biosynthesis | 4C6X | 0.03041 |  | Pro201, NH-Adamantyl | 1.81 |  |
|  |  |  |  |  | 135.00916 | Val278, NH-Aniline | 1.83 | [7] |
|  |  |  |  |  |  | Thr313, O-aryl | 2.08 |  |
| Rv2246 | ß-ketoacyl acyl carrier protein synthase (KasB) | Mycolic acid biosynthesis | 2GP6 | 19.90844 |  | Val278, NH-Adamantyl | 1.90 | [8] |
|  |  |  |  |  | 12.83160 | None |  |  |
| Rv1483 | ß-ketoacyl-ACP reductase (MabA)^b^ | Mycolic acid biosynthesis | 1UZN | - | - |  |  | [9] |
| Rv0635/6 | (3R)-hydroxyacyl-ACP dehydratase (HadAB) | Mycolic acid biosynthesis | 7SVT | - | 20.41328 | Thr140, NH-Adamantyl | 1.98 | [10] |
| Rv0636/7 | (3R)-hydroxyacyl-ACP dehydratase (HadBC) | Mycolic acid biosynthesis | 5ZY8 | - | - |  |  | [11] |
| Rv1484 | Enyol-ACP-reductase, (InhA)^b^ | Mycolic acid biosynthesis | 6R9W | - | - |  |  | [12] |
| Rv0503c | Mycolic acid cyclopropane synthase (CmaA2)^b^ | Mycolic acid biosynthesis | 1KPI | 15.37425 |  | Tyr24, O-aryl | 1.79 | [13] |
|  |  |  |  |  | 42.51670 | Tyr24, O-aryl | 1.89 |  |
| Rv3800c | Polyketide synthase (Pks13) KS Domain^b^ | Mycolic acid biosynthesis | 9F48 | - | - |  |  | [14] |
|  | Polyketide synthase (Pks13) AT Domain |  | 9F48 | - | - |  |  |  |
|  | Polyketide synthase (Pks13) TE Domain |  | 5V40 | - | 54.13346 | Asn1640, O-aryl | 2.06 | [15] |
|  |  |  |  |  |  | Gln1633, NH-Adamantyl | 2.00 |  |
| Rv0206c | Trehalose monomycolate RND transporter (MmpL3) | Mycolic acid transport | 6AJG | - | 0.00045 | Asp256, NH-Aniline | 2.00 | [16] |
|  |  |  |  |  |  | Tyr646, NH-Aniline | 2.82 |  |
| Rv0905 | Enoyl-CoA hydratase 6 (EchA6)^b^ | Mycolic acid biosynthesis | 5DUF | 87.48802 |  | None |  | [17] |
|  |  |  |  |  | 409.85166 | Gln107, NH-Aniline | 2.17 |  |
| Rv3855 | Transcriptional repressor of EthA monooxygenase (EthR)^b^ | Mycolic acid biosynthesis (indirect) | 5EYR | 73.54794 |  | Thr149, NH-Adamantyl | 1.97 | [18] |
|  |  |  |  |  | 0.09363 | Asn176, O-aryl | 2.1 |  |
| Rv3423c | Alanine racemase (alr)^b^ | Peptidoglycan biosynthesis | 1XFC | - | - |  |  | [19] |
| Rv2158c | MurE (Mur Ligase family)^b^ | Peptidoglycan biosynthesis | 2WTZ | - | - |  |  | [20] |
| Rv1018c | Bifunctional enzyme (GlmU)^b^ | Cell wall biosynthesis | 2QKX | 157.40344 | - | Ser112, NH-Adamantyl | 1.79 | [21] |
|  |  |  |  |  |  | Gln83, O-aryl | 2.06 |  |
| Rv1131 | 2-methylcitrate synthase (PrpC)^b^ | Fatty Acid Biosynthesis | 3HWK | - | - |  |  | [22] |
| Rv2214c | Epoxide hydrolase (EphD) | Fatty Acid Biosynthesis | AF-A0A375Z0Y9-F1-v4 | - | - |  |  |  |
| Rv2572c | Aspartyl-tRNA Synthetase (AspS)^b^ | Protein synthesis | 5W25 | - | - |  |  | [23] |
| Rv0041 | Leucyl-tRNA synthase (LeuS)^b^ | Protein synthesis | 5AGS | - | - |  |  | [24] |
| Rv0014c | Protein kinase B (PknB)^b^ | Signal transduction | 5U94 | - | - |  |  | [25] |
| Rv0015c | Protein kinase A (PknA)^b^ | Signal transduction | 6B2Q | - | 349.58518 | Gly145, NH-Adamantyl | 1.83 | [26] |
|  |  |  |  |  |  | Asn146, NH-Aniline | 1.81 |  |
| Rv1092c | Pantothenate kinase (PanK, type 1, CoaA)^b^ | Coenzyme A biosynthesis | 4BFZ | 415.47442 | - | His179, NH-Adamantyl |  | [27] |
| Rv1568 | 3-pyridoxal phosphate (PLP)-dependent aminotransferase (BioA)^b^ | Biotin biosynthesis | 4XJO | 23.87973 |  | Gly172, NH-Adamantyl | 1.82 | [28] |
|  |  |  |  |  | 338.60986 | Gly172, NH-Adamantyl |  |  |
| Rv3722c | Aspartate aminotransferase (aspAT)^b^ | Asp biosynthesis | 6U7A | - | - |  |  | [29] |

a, Lower boundary for estimated affinity [nM] predicted by SeeSAR. b, *Mtb* protein targets identified by Deb et al.

1. Stover, C.K., et al., *New Use of Bcg for Recombinant Vaccines.* Nature, 1991. **351**(6326): p. 456-460.

2. Abrahams, K.A., et al., *Identification of KasA as the cellular target of an anti-tubercular scaffold.* Nat Commun, 2016. **7**: p. 12581.

3. Vilcheze, C., et al., *Rational Design of Biosafety Level 2-Approved, Multidrug-Resistant Strains of Mycobacterium tuberculosis through Nutrient Auxotrophy.* Mbio, 2018. **9**(3).

4. Deb, P.K., et al., *Anti-TB properties, target validation, molecular docking and dynamics studies of substituted 1,2,4-oxadiazole analogues against Mycobacterium tuberculosis.* Journal of Enzyme Inhibition and Medicinal Chemistry, 2021. **36**(1): p. 869-884.

5. Neres, J., et al., *2-Carboxyquinoxalines kill mycobacterium tuberculosis through noncovalent inhibition of DprE1.* ACS Chem Biol, 2015. **10**(3): p. 705-14.

6. Brown, A.K., et al., *Probing the mechanism of the Mycobacterium tuberculosis beta-ketoacyl-acyl carrier protein synthase III mtFabH: factors influencing catalysis and substrate specificity.* J Biol Chem, 2005. **280**(37): p. 32539-47.

7. Schiebel, J., et al., *Structural basis for the recognition of mycolic acid precursors by KasA, a condensing enzyme and drug target from Mycobacterium tuberculosis.* J Biol Chem, 2013. **288**(47): p. 34190-34204.

8. Sridharan, S., et al., *X-ray crystal structure of β-ketoacyl acyl carrier protein synthase II (KasB).* Journal of Molecular Biology, 2007. **366**(2): p. 469-480.

9. Cohen-Gonsaud, M., et al., *Crystal structure of MabA from Mycobacterium tuberculosis, a reductase involved in long-chain fatty acid biosynthesis.* Journal of Molecular Biology, 2002. **320**(2): p. 249-261.

10. Singh, V., et al., *1,3-Diarylpyrazolyl-acylsulfonamides Target HadAB/BC Complex in Mycobacterium tuberculosis.* Acs Infectious Diseases, 2022. **8**(11): p. 2315-2326.

11. Singh, B.K., et al., *The C-terminal end of mycobacterial HadBC regulates AcpM interaction during the FAS-II pathway: a structural perspective.* Febs Journal, 2022. **289**(16): p. 4963-4980.

12. Kamsri, P., et al., *Discovery of New and Potent InhA Inhibitors as Antituberculosis Agents: Structure-Based Virtual Screening Validated by Biological Assays and X-ray Crystallography.* Journal of Chemical Information and Modeling, 2020. **60**(1): p. 226-234.

13. Huang, C.C., et al., *Crystal structures of mycolic acid cyclopropane synthases from.* Journal of Biological Chemistry, 2002. **277**(13): p. 11559-11569.

14. Johnston, H.E., et al., *Cryo-electron microscopy structure of the di-domain core of Mycobacterium tuberculosis polyketide synthase 13, essential for mycobacterial mycolic acid synthesis.* Microbiology (Reading), 2024. **170**(10).

15. Aggarwal, A., et al., *Development of a Novel Lead that Targets M. tuberculosis Polyketide Synthase 13.* Cell, 2017. **170**(2): p. 249-+.

16. Zhang, B., et al., *Crystal Structures of Membrane Transporter MmpL3, an Anti-TB Drug Target.* Cell, 2019. **176**(3): p. 636-+.

17. Cox, J.A.G., et al., *THPP target assignment reveals EchA6 as an essential fatty acid shuttle in mycobacteria.* Nature Microbiology, 2016. **1**(2).

18. Nikiforov, P.O., et al., *A fragment merging approach towards the development of small molecule inhibitors of EthR for use as ethionamide boosters.* Organic & Biomolecular Chemistry, 2016. **14**(7): p. 2318-2326.

19. LeMagueres, P., et al., *The 1.9 Å crystal structure of alanine racemase from Mycobacterium tuberculosis contains a conserved entryway into the active site.* Biochemistry, 2005. **44**(5): p. 1471-1481.

20. Basavannacharya, C., et al., *ATP-dependent MurE ligase in Mycobacterium tuberculosis: Biochemical and structural characterisation.* Tuberculosis, 2010. **90**(1): p. 16-24.

21. Zhang, Z., et al., *Structure and function of GlmU from.* Acta Crystallographica Section D-Structural Biology, 2009. **65**: p. 275-283.

22. Baugh, L., et al., *Increasing the structural coverage of tuberculosis drug targets.* Tuberculosis, 2015. **95**(2): p. 142-148.

23. Gurcha, S.S., et al., *Biochemical and Structural Characterization of Mycobacterial Aspartyl-tRNA Synthetase AspS, a Promising TB Drug Target.* Plos One, 2014. **9**(11).

24. Palencia, A., et al., *Discovery of Novel Oral Protein Synthesis Inhibitors of Mycobacterium tuberculosis That Target Leucyl-tRNA Synthetase.* Antimicrobial Agents and Chemotherapy, 2016. **60**(10): p. 6271-6280.

25. Wlodarchak, N., et al., *In Silico Screen and Structural Analysis Identifies Bacterial Kinase Inhibitors which Act with β-Lactams To Inhibit Mycobacterial Growth.* Molecular Pharmaceutics, 2018. **15**(11): p. 5410-5426.

26. Wang, T.S., et al., *Mtb PKNA/PKNB Dual Inhibition Provides Selectivity Advantages for Inhibitor Design To Minimize Host Kinase Interactions.* Acs Medicinal Chemistry Letters, 2017. **8**(12): p. 1224-1229.

27. Bjorkelid, C., et al., *Structural and Biochemical Characterization of Compounds Inhibiting Mycobacterium tuberculosis Pantothenate Kinase.* Journal of Biological Chemistry, 2013. **288**(25): p. 18260-18270.

28. Liu, F., et al., *Structure-Based Optimization of Pyridoxal 5′-Phosphate-Dependent Transaminase Enzyme (BioA) Inhibitors that Target Biotin Biosynthesis in.* Journal of Medicinal Chemistry, 2017. **60**(13): p. 5507-5520.

29. Jansen, R.S., et al., *Aspartate aminotransferase Rv3722c governs aspartate-dependent nitrogen metabolism in.* Nature Communications, 2020. **11**(1).
